# Inference of the *Xenopus tropicalis* embryonic regulatory network and spatial gene expression patterns

**DOI:** 10.1186/1752-0509-8-3

**Published:** 2014-01-08

**Authors:** Zhenzhen Zheng, Scott Christley, William T Chiu, Ira L Blitz, Xiaohui Xie, Ken WY Cho, Qing Nie

**Affiliations:** 1 Department of Mathematics, University of California, Irvine, CA 92697, USA; 2 Department of Developmental and Cell Biology, University of California, Irvine, CA 92697, USA; 3 Department of Computer Science, University of California, Irvine, CA 92697, USA; 4 Center for Complex Biological Systems, University of California, Irvine, CA 92697, USA; 5 Center for Mathematical and Computational Biology, University of California, Irvine, CA 92697, USA; 6 Department of Surgery, University of Chicago, Chicago, IL 60637, USA; 7 Beijing Computational Science Research Center Beijing, China

## Abstract

**Background:**

During embryogenesis, signaling molecules produced by one cell population direct gene regulatory changes in neighboring cells and influence their developmental fates and spatial organization. One of the earliest events in the development of the vertebrate embryo is the establishment of three germ layers, consisting of the ectoderm, mesoderm and endoderm. Attempts to measure gene expression *in vivo* in different germ layers and cell types are typically complicated by the heterogeneity of cell types within biological samples (i.e., embryos), as the responses of individual cell types are intermingled into an aggregate observation of heterogeneous cell types. Here, we propose a novel method to elucidate gene regulatory circuits from these aggregate measurements in embryos of the frog *Xenopus tropicalis* using gene network inference algorithms and then test the ability of the inferred networks to predict spatial gene expression patterns.

**Results:**

We use two inference models with different underlying assumptions that incorporate existing network information, an ODE model for steady-state data and a Markov model for time series data, and contrast the performance of the two models. We apply our method to both control and knockdown embryos at multiple time points to reconstruct the core mesoderm and endoderm regulatory circuits. Those inferred networks are then used in combination with known dorsal-ventral spatial expression patterns of a subset of genes to predict spatial expression patterns for other genes. Both models are able to predict spatial expression patterns for some of the core mesoderm and endoderm genes, but interestingly of different gene subsets, suggesting that neither model is sufficient to recapitulate all of the spatial patterns, yet they are complementary for the patterns that they do capture.

**Conclusion:**

The presented methodology of gene network inference combined with spatial pattern prediction provides an additional layer of validation to elucidate the regulatory circuits controlling the spatial-temporal dynamics in embryonic development.

## Background

Detailed gene regulatory networks (GRNs) in a number of invertebrate species have provided an unprecedented global overview of the genetic program controlling development in sea urchin, *Drosophila*, and *C. elegans*[1-4] and have revealed a number of important and conserved regulatory cassettes employed in a diversity of developmental contexts [5]. While generation of such networks will be also extremely valuable in understanding the mechanisms governing cell fate specification in vertebrate systems, similar work in vertebrates is challenging as the number of cell types, genome organization and genes involved in regulating the biological processes are significantly more complex.

In all triploblastic metazoans, establishment of the primary germ layers (endoderm, mesoderm and ectoderm) occurs early, during blastula and gastrula stages. In the *Xenopus* blastula the presumptive germ layers are arranged along the vegetal-animal axis with endoderm arising from the vegetal cells, mesoderm in an equatorial ring and the ectoderm on the top overlying the blastocoel cavity. This simple spatial arrangement in developing embryos, taken together with a low complexity in terms of numbers of different cell types and the ease in manipulating gene expression, makes the amphibian *Xenopus* ideally suited to study GRNs in early vertebrate development.

*Xenopus* developmental biologists have spent nearly 20 years in generating a prototype GRN describing the mesendoderm [6,7]. Despite this effort, these GRN diagrams are very incomplete and provide only a limited preview of the *in vivo* condition. New alternative approaches are urgently needed to generate GRNs that incorporate more genes and have predictive features. In this paper, we present a novel method to elucidate gene regulatory circuits from aggregate gene expression measurements in embryos of the frog *Xenopus tropicalis* using gene network inference algorithms and then test the ability of the inferred networks to predict spatial gene expression patterns.

The primary methodologies for gene network inference include probabilistic graphical models [8-11], information-theoretic approaches [12,13], ordinary differential equations (ODEs) (among which include linear ODEs for steady-state data [14-17], linear ODEs for time series data [15,16,18-21] and nonlinear ODEs for time series data that adopt heuristic search strategies [22-26]) and linear regression models [11,20,27,28]. There are numerous reviews of these methods and other approaches [29-34].

In this work, we examine gene expression profile changes of hundreds of genes at several developmental stages after loss-of-function analyses. We then employ two inference models with different underlying assumptions, a linear ODE model for steady-state data and a linear Markov model for time series data, to elucidate the core dorsal mesoderm and endoderm regulatory circuits. Both models incorporate sparseness control on the network connections and prior network information, and they can be solved with the same optimization framework. Using one inferred network in combination with known dorsal-ventral expression pattern images of a subset of genes, we define an optimization problem to predict spatial patterns for all genes in the network. The spatial pattern prediction provides an additional layer of validation for the regulatory circuits controlling the spatial-temporal dynamics in embryonic development.

We model the gene network using ordinary differential equations (ODEs) that describe gene regulation as a function of other genes:

$$\frac{{\mathrm{dx}}_{i}\left(t\right)}{\mathrm{dt}}=F_{i}\left(x_{1}\left(t\right),\ldots ,x_{p}\left(t\right)\right)$$

where *x*_*i*_(*t*) is the concentration of mRNA for gene *i* measured at time *t*, *dx*_*i*_(*t*)/*dt* is the rate of change for the mRNA concentration of gene *i*, and *p* is the number of genes. Each function *F*_*i*_ represents all of the various processes and factors that affect the amount of mRNA for gene *i*. Previously, we presented a linear steady-state ODE model for gene network inference that incorporates regularization terms for sparseness and prior network information [17]. We showed that inclusion of prior knowledge about the network structure in the inference process increased performance, that incorrect connections in network structure knowledge did not hurt performance, and that a mixture of correct and incorrect connections given as prior knowledge performed better than giving no prior network information.

We employ our steady-state ODE model to gene expression data from the *Xenopus* embryo. Since the linear steady-state ODE model assumes that observations are made when the experimental system is at a steady-state equilibrium, the model cannot directly incorporate temporal dynamics for the multiple developmental stages present in our data. One technique to account for such dynamics is to approximate the derivatives for the variables (i. e., *dx*_*i*_(*t*)/*dt*), but this approximation can be inaccurate for the long time intervals typical in biological data. An alternative approach proposed by Linde et al. [21,35] is to consider a first-order Markov model where the gene expression at time *k* is a linear function of its regulators at the previous time *k*–1, i.e., $x_{i}^{k}=\sum_{j=1}^{p}W_{\mathrm{ij}}x_{j}^{k-1}$, and *W* is the linear gene interaction matrix. However, this model suffers from an issue typical of gene network inference models, which is that the number of genes is greater than the number of experimental observations. Therefore, the system is underdetermined and the model tends to produce a dense gene network that overfits the data. Linde et al. utilize a heuristic search strategy to produce sparse networks, however it is not integrated into the optimization problem and thus it is hard to gauge the effectiveness of the heuristic [21,35]. Various regularization techniques, which are integrated into the optimization problem, have been introduced to prevent overfitting and to perform variable selection including ridge regression [36], LASSO [37-39], and elastic net [40]. Ridge regression tends to achieve better prediction performance through a bias-variance tradeoff among all the variables, while LASSO specifically enforces sparseness by excluding poor predictor variables, and elastic net combines the two techniques. In our prior work, we applied LASSO in our linear steady-state ODE model to produce a parsimonious regulatory network that is optimal as tested by cross-validation, and we showed how the LASSO regularization operator could be extended to incorporate prior network information [17]. In this paper, we extend the Markov model to include regularization terms that enforce sparseness of the inferred gene network and allow incorporation of prior network information. We apply the model to simulated data from test networks and present results on the model’s ability to recover the network from differing number of observations and mixtures of correct and incorrect connections provided as prior network information. We apply both models to the aggregate gene expression data of the heterogeneous cell types in the *Xenopus* embryo, and then compare the ability of each model to recover the core regulatory circuits.

Advances in bioimaging and image analysis are allowing gene expression data to be mapped and studied within a spatial context for organisms and tissue [41-45]. This has led to the recognition and challenge of using spatial gene expression data to reconstruct the regulatory circuits responsible for those spatial patterns, such as in a recent case study of reverse engineering the well-studied gap gene network responsible for segmentation in the embryo of *D. melanogaster*[46-49]. Our research is the first attempt to our knowledge to apply similar techniques for *Xenopus*. One of the challenges is quantifying gene expression from spatial pattern images [50], however we take a simpler approach by categorizing the spatial pattern based upon the assessment of a biological expert. Given a set of spatial gene expression image obtained from Xenbase [51], we transform the expression along the dorsal-ventral axis of the embryo into a one-dimensional representation. We then define an optimization problem that takes an inferred gene network, either from the steady-state ODE or Markov model, and a subset of spatial data to predict the spatial patterns for the remaining genes. We characterize the performance for each model in their ability to predict the spatial expression for genes with known patterns, and we discuss hypothesized spatial patterns for genes where no such data exists. Our approach suggests that a single modeling method is not sufficient to capture all aspects of spatial gene expressions, and the differences in the underlying assumptions for each model may provide insights about the spatial-temporal dynamics in embryonic development.

## Results and discussion

### Simulation results

We generated a set of time series simulation data to test the Markov model. Five random networks containing *p* = 10 nodes with 2–3 uniform randomly selected incoming edges were generated for a total of exactly 25 edges in the network; each edge had a weight drawn from the normal distribution *N* (0, 100). A large variance was used to avoid generating simulation data with big values, described in more detail below. Each network was verified to be nonsingular.

For each random network, we generated 15 observations where each observation contained 4 consecutive time points (i.e., *k*_max_ = 3 in (Eq. 1.3)). Specifically, the time series data *x*^*k*^ in each observation were generated as following: *y*^4^ was randomly drawn from the standard normal distribution, *x*^4^ was generated by adding noise to *y*^4^, i.e., *x*^4^ = *y*^4^ + *N* (0,0.3), then

$$\begin{array}{l} x^{k}=W^{-1}\left(y^{k+1}+N\left(0,0.1\right)\right)+N\left(0,0.3\right),\\ y^{k}=W^{-1}y^{k+1}, \end{array}$$

where *k* = 3, 2, 1, *x*^*k*^ represents the *k* th time-point data in one observation and *y*^*k*^ represents the *k* th time-point ideal data without noise in one observation. Here, two kinds of noise were added: the intrinsic noise (e.g., stochastic fluctuations in the underlying biological process) was drawn from *N* (0,0.1) and the extrinsic noise (e.g., measurement errors) was drawn from *N* (0,0.3). The weight of each edge in the randomly generated networks was drawn from *N* (0, 100). The large variance can help avoid generating data with large values that can skew the inference process and produce numerical errors. Using a small variance tends to generate large values in *W*^–1^, thus each time point will produce increasingly larger values for *y*^*k*^ and *x*^*k*^. The generated random networks and time series data used in producing the simulation results are provided in Additional file 1.

For our experiments that utilize existing network information, we provide a Boolean matrix *W*^0^, where an entry $W_{\mathrm{ij}}^{0}=0$ indicates a directed interaction from gene *j* to gene *i*, while $W_{\mathrm{ij}}^{0}=1$ for all other edges.

Using leave-one-out cross-validation, we find the values for the regularization parameters, *α* (sparsity) and *β* (prior network), for each gene that minimizes the cross-validation error. A proportional error is calculated to measure the algorithm’s performance throughout this section. Since we introduce noise into the simulated data, the cross-validation error will vary with the number of observations. Therefore, in each simulation run we divide the minimal cross-validation error by the minimal least-squares error obtained using linear regression without any regularization terms. This normalizes the error relative to the minimal possible error achievable through linear regression. Then we take an average across all the random networks to produce the final proportional error.

#### Inference error decreases as the number of observations increases

**Figure 1:**
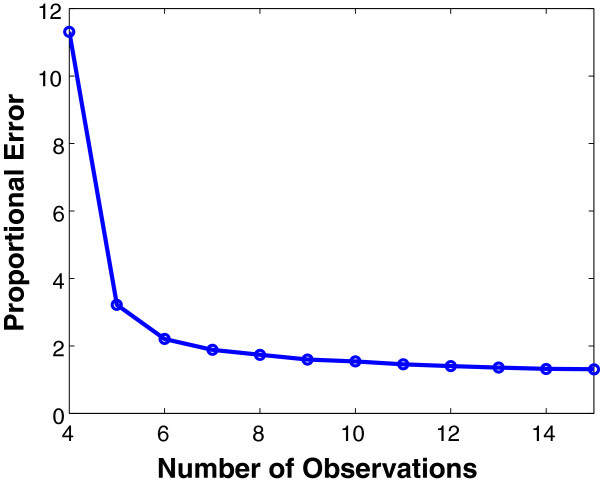
**Inference error versus number of observations.** The proportional error (i.e., inference error) denotes the minimal cross-validation error divided by the minimal least-squares error of the linear regression without any regularization terms and averaged over five random networks. The proportional errors decrease with more observations and stabilize when there are enough observations.

We first examined the effect of the number of observations on the prediction performance (Figure 1). As would be expected, as more observations are provided to the inference algorithm, performance improves and the error approaches the minimal possible error achievable through linear regression. More observations serve to provide better estimates for the edge weights in each random network.

#### Providing valid edges as prior network information increases performance

**Figure 2:**
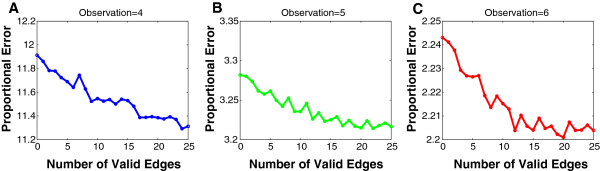
**Inference error versus number of random valid edges provided.** The proportional error (i.e., inference error) denotes the minimal cross-validation error divided by the minimal least-squares error of the linear regression without any regularization terms and averaged over five random networks and five random sets of valid edges. Zero to 25 (i.e., the number of all edges in each random network) prior edges are provided respectively. **(A)**, **(B)** and **(C)** are the results of 4, 5 and 6 observations, respectively. The proportional errors decrease when more valid edges are provided. Prior connections appear to be more important when only a few observations are available.

Although providing more observations will increase prediction performance, only relatively few observations are usually available compared to the large number of genes. Here, we demonstrated that providing existing edges could enhance prediction performance especially in the situation of few observations. Zero to twenty-five (i.e., the number of all edges in each random network) randomly chosen prior edges were provided respectively to the inference algorithm (Figure 2). For a fixed number of valid edges, we generated five random networks and five sets of random valid edges for each network. The proportional errors were averaged over the five networks and five sets of valid edges. It was found that the errors decrease when more valid edges were provided. This effect was related to the number of observations and prior connections appeared to be more important when only a few observations were available.

#### Providing invalid edges as prior network information does not affect performance

**Figure 3:**
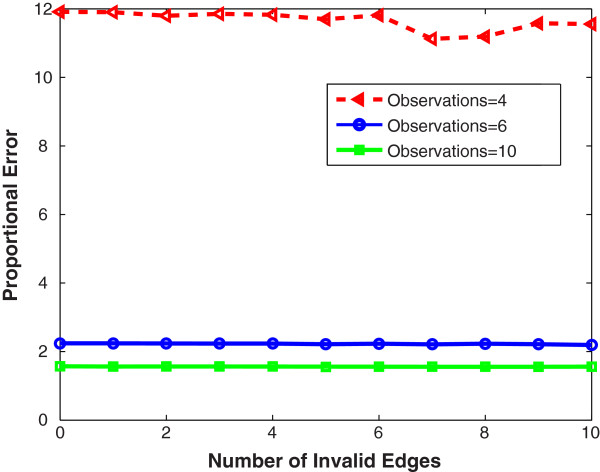
**Inference error versus number of random invalid edges (without valid edges) provided.** The proportional error (i.e., inference error) denotes the minimal cross-validation error divided by the minimal least-squares error of the linear regression without any regularization terms and averaged over five random networks. The red, blue and green curves represent 4, 6 and 10 observations, respectively. Providing invalid network edges only has little effect on the errors, especially when many observations are available. The errors become smaller as the number of observations increases, which is consistent with the results in Figure 1.

As a contrast, we also examined the effect of providing incorrect edges. Zero to ten incorrect edges were randomly chosen respectively as the prior information (Figure 3). Providing invalid network edges only (without valid edges) had little effect on the errors, especially when many observations were available. The reason is that if the invalid edges do not help to reduce the minimal cross-validation error, the prior network information will be ignored [17]. The errors became smaller as the number of observations increased, which was consistent with the results in Figure 1.

#### Consistent performance is maintained with a mixture of valid and invalid edges in prior network information

**Figure 4:**
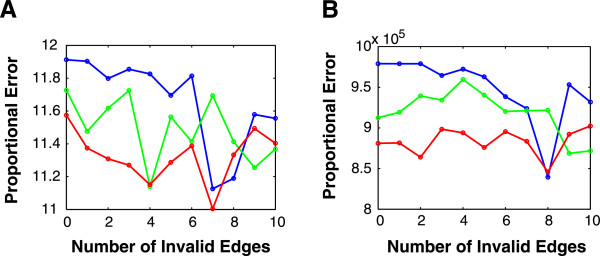
**Inference error versus number of random invalid edges.** The proportional error (i.e., inference error) denotes the minimal cross-validation error divided by the minimal least-squares error of the linear regression without any regularization terms and averaged over five random networks. The blue, green and red curves represent 0, 5 and 20 random valid edges, respectively. 4 and 2 observations are considered in **(A)** and **(B)**, respectively. When there are more valid edges (e.g., valid = 20), the errors are generally smaller as a whole. When only a few observations are available, the valid edges appear to be even more important. The cross-validation errors are in a large scale (i.e., 10^5^) in **(B)**, because they are divided by the least-squares errors, while fewer observations are easier to be over fitted with small least-squares errors (e.g., 10^-6^ ~ 10^-4^).

Since the prior network information may contain both valid and invalid edges, it is important to examine the effect of providing both kinds of edges on network performance. The proportional errors were averaged over five random networks with randomly chosen mixed edges (Figure 4). We observed that when there were more valid edges (e.g., valid = 20), the errors were generally smaller as a whole. Even when the valid edges were mixed with invalid edges, the errors did not become much larger. It was probable to achieve smaller errors with invalid edges than without invalid edges. However, this is not very surprising. For example, considering an extreme case in which all the valid edges are provided and all the other edges are chosen to be invalid edges, then *W*^0^ = 0, i.e., there are no prior edges to be punished in the optimization problem. As a consequence the least-squares is easier to be over fitted and smaller cross-validation errors are easier to be produced. Comparing Figures 4A and 4B, we could also see that the effect of valid edges was related with the number of observations. When there are only a few observations available, the valid edges appear to be even more important, e.g., the curves in Figure 4B separate more with each other than those in Figure 4A. Based on the observation, we hypothesize that if many observations are available, the effect of valid edges on the errors will be weakened.

#### Comparison of the ODE model and the Markov model

Based on the above observations, three common conclusions can be obtained from the ODE model (Eq. 1.2) and the Markov model (Eq. 1.3): (1) the proportional errors decrease as the number of observations increases; (2) providing invalid edges alone does not affect the prediction performance; (3) providing valid edges is generally helpful to improve the performance especially when only a few observations are available. The difference is that the ODE simulation data [17] is separated into two groups and their noise is drawn from *N* (0,0.3) and *N* (0,0.1) respectively, while all the Markov simulation data contains both the above noise simultaneously which generates larger noise in the above simulations and weakens the effect of prior valid edges.

### Inference of *Xenopus tropicalis* embryonic regulatory network

**Figure 5:**
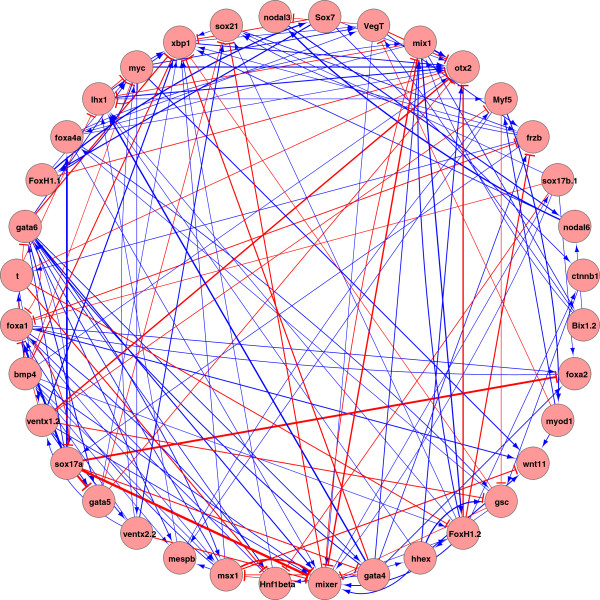
**Inferred network from the linear ODE model.** Positive interactions are shown in blue and negative interactions are shown in red. The strength of connections is indicated via the thickness of lines connecting the genes. The interactions vary from −1.2921 to 1.4132 and a threshold 0.25 is applied, i.e., all the edges with the weights smaller than 0.25 are discarded.

**Figure 6:**
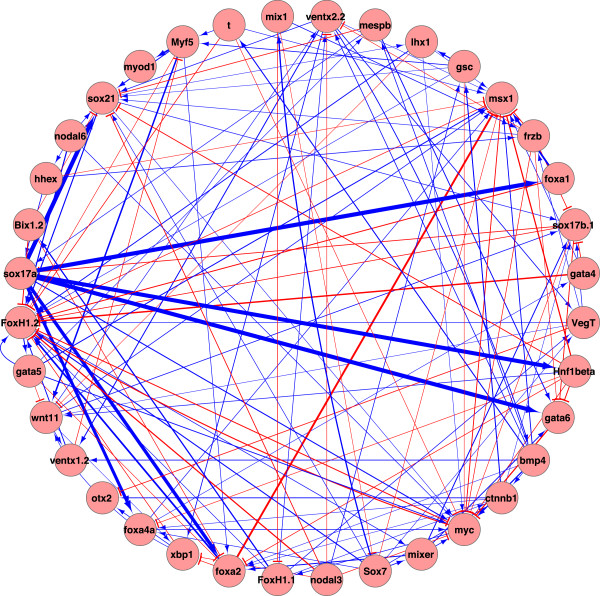
**Inferred network from the linear Markov model.** Positive interactions are shown in blue and negative interactions are shown in red. The strength of connections is indicated via the thickness of lines connecting the genes. The interactions vary from −1.1317 to 2.5607 and a threshold 0.2 is applied, i.e., all the edges with the weights smaller than 0.2 are discarded.

We generated NanoString probes for 177 *Xenopus tropicalis* genes (see Additional file 2), choosing to target mostly transcription factors and secreted signaling factors that are expressed in early embryos as these are important developmental control genes. We performed four morpholino antisense oligonucleotide (MO) experiments to knockdown the expression of vegt, sox17, ctnnb1 (β-catenin) and foxh1 proteins in *X. tropicalis*. Total RNA was isolated from embryos at four different developmental stages (blastula stage 9 and gastrula stages 10, 11 and 12.5) and subjected to gene expression profiling analysis using the Nanostring nCounter system. Experiments were repeated three times to obtain biologically independent data sets. The NanoString data from the MO experiments is provided as additional files (see Additional files 3 and 4). Reproducibility of Nanostring data in these triplicate samples showed R^2^ = 0.98, indicating that the data are of high quality and reproducible (data not shown). The expression data for each gene is normalized by its maximum expression data. Since the time interval is relatively long (i.e., in hours instead of minutes), we assumed this process as a dynamic equilibrium and used the steady-state ODE model (Eq. 1.2) and Markov model (Eq. 1.3) to infer gene regulatory networks. As a balance of the amount of available data and the size of the to-be-inferred network, we chose 36 out of 177 genes to infer the interactions between the 36 genes. 36 genes were chosen for analyses because of the availability of their spatiotemporal expression patterns and known transcriptional activities [7]. In addition, there were 46 prior gene interactions available for us [7]. The inferred network from the ODE model and Markov model are in Figures 5 and 6, respectively. Table 1 lists the prior connections and the connections in the inferred ODE network and Markov network which were consistent with the prior connections.

**Table 1:** The 46 prior connections and the connections inferred from the linear ODE model and Markov model

**Prior information**	**Linear ODE model**	**Linear Markov model**
sox17a regulates hnf1b	Inferred	Inferred
sox17a regulates foxa4a	Inferred	Inferred
sox17a regulates foxa1	Inferred	Inferred
sox17a regulates foxa2	Inferred	Inferred
sox17a regulates gata4	Inferred	
sox17a regulates gata5	Inferred	
sox17a regulates gata6	Inferred	Inferred
sox17a regulates bix1.2	Inferred	
sox17b.1 regulates hnf1b	Inferred	Inferred
sox17b.1 regulates foxa4a		Inferred
sox17b.1 regulates foxa1	Inferred	Inferred
sox17b.1 regulates foxa2	Inferred	Inferred
sox17b.1 regulates gata4	Inferred	
sox17b.1 regulates gata5	Inferred	Inferred
sox17b.1 regulates gata6	Inferred	Inferred
sox17b.1 regulates bix1.2	Inferred	Inferred
sox7 regulates sox17a		
sox7 regulates sox17b.1		
gata4 regulates sox17a		
gata4 regulates sox17b.1		Inferred
gata5 regulates sox17a		Inferred
gata5 regulates sox17b.1	Inferred	Inferred
gata6 regulates sox17a	Inferred	
gata6 regulates sox17b.1		
bix1.2 regulates sox17a	Inferred	Inferred
bix1.2 regulates sox17b.1	Inferred	
vegt regulates mix1	Inferred	
vegt regulates mixer	Inferred	
vegt regulates sox17a		Inferred
vegt regulates sox17b.1	Inferred	Inferred
foxh1 regulates otx2	Inferred	Inferred
foxh1 regulates lhx1	Inferred	Inferred
foxh1 regulates mix1	Inferred	Inferred
foxh1 regulates mixer	Inferred	Inferred
foxh1 regulates bix1.2	Inferred	Inferred
foxh1 regulates t		
foxh1 regulates ventx2.2		Inferred
foxh1 regulates sox17a	Inferred	Inferred
foxh1 regulates sox17b.1	Inferred	
foxh1 regulates frzb	Inferred	Inferred
foxh1 regulates gsc		Inferred
foxh1 regulates hhex	Inferred	Inferred
foxh1 regulates msx1	Inferred	Inferred
ventx2.2 regulates ventx1.2	Inferred	Inferred
ventx1.2 regulates myf5	Inferred	Inferred
t regulates myf5		Inferred

The blank stands for missing edges in the inferred networks. There are 34 and 32 connections same as the prior information in the networks inferred from the linear ODE model and Markov model, respectively. Among these connections, 25 connections are shared. Note that the prior information we provide to the algorithm is only the existence/non-existence of interactions, not including their information about activation or inhibition.

The inferred network from the ODE model contains 694 edges and 34 out of 46 (p-value = 0.006) prior connections are correctly inferred. The full list of inferred connections from the ODE model are in Additional file 1: Table S1. The inferred network from the Markov model contains 410 edges and 32 (p-value = 0) edges are consistent with the prior information. The full list of inferred connections from the Markov model are in Additional file 1: Table S2. Details for p-value calculations are provided in Additional file 1. 25 connections are shared among the connections in the inferred ODE network and Markov network (details are in Table 1). The Markov network is more sparse than the ODE network, e.g., the average degree of all nodes is 11.39 for the Markov network and 19.28 for the ODE network. There are 172 common connections among all the connections in both networks.

The cross-validation procedure calculates values for the regularization parameters, *α* (sparsity) and *β* (prior network) such that the cross-validation error is minimized. However, the resultant network does not necessarily contain all of the connections provided in the prior network as with our study where 34 (ODE) and 32 (Markov) of the 46 prior networks connections are in the inferred network. This can occur for a number of reasons, for example: 1) the prior network information may be incorrect and thus excluded, 2) the experimental data may lack a discriminatory signal that the algorithm can use to infer the connection, 3) the cross-validation error may be too stringent by excluding connections with minimal support, or 4) the non-linear dynamics of the prior network connection may not be sufficiently captured by the linear model. If cross-validation for the sparsity control parameter *α* in (Eqs. 1.2 and 1.3) is not used, *α* can be varied to produce more or less prior network connections. In the ODE model, by setting *α = 0.006* then 42 prior network connections are obtained, while *α = 0.0* provides all 46 prior connections. Likewise for the Markov model, setting *α = 0.0117* provides 35 prior connections, *α = 0.008* provides 40 prior connections, and *α = 0.0* provides all 46 prior connections. However, for such cases the cross-validation errors are not as good as the one obtained through the learning algorithm on the sparsity parameter. For example, the cross-validation errors are 0.5179, 0.5162 and 0.5129 respectively in the above three settings of *α* for the Markov model, while the optimal cross-validation error we obtained was 0.5014. Furthermore, increasing the value of the sparsity control parameter may decrease the number of prior connections by enforcing more sparsity and eliminating connections that are least consistent with the experimental data. Therefore, the number of prior network connections within the inferred network should not be considered as a strict measure of the accuracy of the algorithm, instead it is a relative indication of the information provided within the experimental data that is consistent with the prior network, while taking into account the trade-off of generalization versus over-fitting by the inference algorithm.

#### Inference of the core dorsal endoderm circuit

Both inference models recovered the core circuitry controlling dorsal endoderm specification including direct regulation of *hnf1β, foxa1, foxa2*, *foxa4a, gata5*, *gata6* and *bix1* by *sox17*; direct or indirect regulation of *gata4* by *sox17*; as well as the direct or indirect regulatory feedback of *gata4-6* and *bix1* onto *sox17*. Both models predicted *vegt* regulation of *sox17*. However, the two models predict the regulation of two *sox17* genes, *sox17a* and *sox17b*, which are paralogs, to be different. The ODE model usually does not differentiate regulatory action of *sox17a* and *sox17b*: both are regulators of *hnf1β, foxa1, foxa2, gata4, gata5, gata6* and *bix1*. The Markov model infers that both *sox17a* and *sox17b* are regulators of *hnf1b, foxa1, foxa2, foxa4a* and *gata6*. However, the Markov model sometimes splits the regulatory action of *sox17a* and *sox17b*: *sox17b* is a direct regulator of *gata5* and *bix1* while *sox17a* is not. Given the differing model assumptions with the ODE model assuming steady-state and the Markov model assuming temporal change, the predictions could suggest that *sox17a* and *sox17b* have different temporal actions in the context of the feedback loop with *gata4-6* and *bix1*[52], even though *sox17a* and *sox17b* are similar in their expression and activity [53]. One hypothesis is that *sox17b* is the primary driver of temporal change for the feedback loop, while *sox17a* stabilizes those changes. Better understanding of the other factors involved in the feedback loop could help resolve this difference.

#### Inference of the core dorsal mesoderm circuit

Both inference models recovered the core circuitry controlling dorsal mesoderm specification with foxh1 being a direct regulator of *mix1, mixer, lhx1, bix1, otx2, sox17, frzb, msx1* and *hhex.* Both models predicted *ventx2* regulation of *ventx1* and *ventx1* regulation of *myf5* in the ventrolateral mesoderm. The Markov model also predicted *foxh1* regulation of *gsc* in the dorsal mesoderm, and *ventx2.2* in the ventrolateral mesoderm. Only the ODE model was able to predict Vegt regulation of *mix1* and *mixer*. Both inference models recovered some core dorsal mesoderm circuit with slightly different gene sets.

### Inference of *Xenopus tropicalis* embryonic spatial gene expression

**Figure 7:**
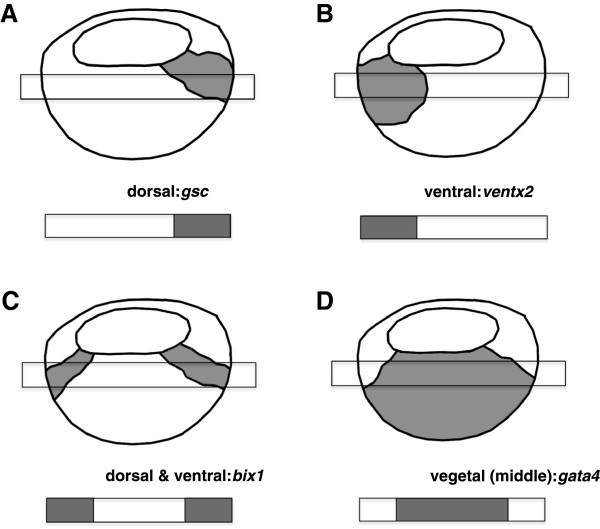
**Spatial gene expression patterns in *Xenopus *embryos. (A)** The *ventx* gene is expressed ventrally. **(B)** The *gsc* gene is expressed dorsally. **(C)** The *bix1* gene is expressed in both the ventral and dorsal regions. **(D)** The *gata4* gene is expressed in the vegetal region. The rectangular bar across the embryo indicates the portion of the image viewed for classification purposes, with the bar under the image showing the 1-dimensional representation of gene expression.

The inferred network can be applied to predict gene spatial patterns. Given known spatial gene expression patterns for some genes, the network and those patterns can be used to predict the unknown patterns for the other genes in the network. The source of publications of the 28 genes with known spatial expression patterns and links to their pictures from Xenbase are included in as additional files (see Additional files 5 and 6). Typical spatial expression patterns of *ventx2*, *gsc, bix1* and *gata4* in *Xenopus* embryos are illustrated in Figure 7. The *ventx* gene is expressed ventrally, *gsc* is expressed dorsally, *bix1* is expressed both ventrally and dorsally, and *gata4* is expressed in the vegetal region. We classified the known expression patterns of 28 genes (shown in the last column in Table 2) among our 36 total genes, as dorsal (d), ventral (v), both dorsal and ventral (b), middle or vegetal (m) and uniformly expressed (u). We used these partial known patterns with the regulatory networks inferred above to predict the expression patterns of all the genes, including unverified patterns. We abstracted the complex embryo by regarding the dorsal-ventral division as a one-dimensional interval, which was further partitioned into three regions. Based on this representation, we defined ODE and Markov spatial prediction models for steady-state data and time series data, respectively.

**Table 2:** Predicted gene spatial patterns based on the Markov spatial prediction model for 36 genes from averages with 1000 random initial patterns

**Gene**	**Predicted pattern**	**Prior pattern**
‘bix1.2’	‘d’	‘b’
‘bmp4’	‘v’	‘v’
‘ctnnb1’	‘v’	‘d’
‘foxa1’	‘u’	‘m’
‘foxa2’	‘v’	‘m’
‘foxa4a’	‘d’	‘b’
‘foxh1’	‘u’	‘u’
‘foxh1.2’	‘v’	[ ]
‘frzb’	‘d’	‘d’
‘gata4’	‘d’	‘m’
‘gata5’	‘u’	‘m’
‘gata6’	‘v’	‘m’
‘gsc’	‘d’	‘d’
‘hhex’	‘u’	‘d’
‘hnf1b’	‘u’	[ ]
‘lhx1’	‘u’	‘d’
‘mespb’	‘v’	[ ]
‘mix1’	‘u’	‘u’
‘mixer’	‘u’	‘m’
‘msx1’	‘v’	‘v’
‘myc’	‘v’	[ ]
‘myf5’	‘u’	[ ]
‘myod1’	‘u’	[ ]
‘nodal3’	‘u’	‘d’
‘nodal6’	‘u’	‘m’
‘otx2’	‘d’	‘d’
‘sox17a’	‘u’	‘m’
‘sox17b.1’	‘u’	‘m’
‘sox21’	‘v’	[ ]
‘Sox7’	‘v’	[ ]
‘t’	‘v’	‘b’
‘vegt’	‘u’	‘u’
‘ventx1.2’	‘v’	‘v’
‘ventx2.2’	‘v’	‘v’
‘wnt11’	‘b’	‘b’
‘xbp1’	‘u’	‘b’

‘d’, ‘v’, ‘b’, ‘u’, ‘m’ stand for dorsal, ventral, both dorsal and ventral, uniform and middle (vegetal) patterns, respectively. ‘[]’ represents unknown patterns.

**Figure 8:**
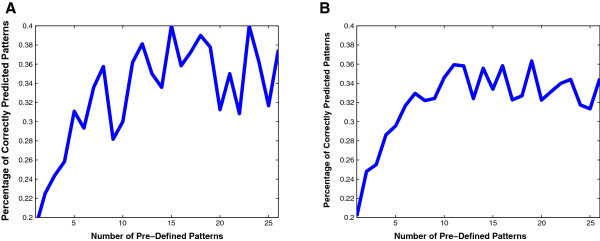
**Percentage of correctly predicted patterns in the ODE spatial prediction model.** Given a fixed number (from 1 to 26) of pre-defined patterns, the percentages are averaged over 20 sets (Figure **(A)**) and 100 sets (Figure **(B)**) of randomly chosen genes with pre-defined patterns. As expected, the percentages of correctly predicted patterns are more stable when they are averaged over more sets of randomly chosen genes. Also, the prediction percentages show an increasing trend as more pre-defined patterns are provided.

We first considered the prediction performance of the ODE spatial prediction model. Our approach is to provide different data sets of known gene expression patterns to train the program, and then to observe the performance of the model in predicting the expression of the remaining genes. In Figure 8 we plotted the percentage of correctly predicted patterns as the number of pre-defined patterns varies. For each fixed number of pre-defined patterns, the percentage was averaged over 20 sets and 100 sets of randomly chosen genes with pre-defined patterns (Figure 8A and B, respectively). As expected, the percentages of correctly predicted patterns are more stable when they are averaged over more sets of randomly chosen genes. Also, the prediction percentages show an increasing trend as more pre-defined patterns are provided.

We sequentially chose expression patterns of all possible combinations of 27 genes out of the total 28 genes and used them as constraints in an optimization problem to predict the remaining gene expression pattern. 11 (i.e., 39.3%) gene expression patterns were correctly predicted based on the 28-gene network inferred from the ODE model. The performance is better than random, which is expected to be 20%. The correctly predicted genes were: *bmp4, foxa1, gata4, gata6, gsc, hhex, lhx1, otx2, sox17b.1, ventx1.2 and ventx2.2.* We also used all the 28 known patterns as constraints to predict the expression patterns of the remaining 8 genes based on the 36-gene network inferred from the ODE model. The predicted patterns are shown in Table 3.

**Table 3:** Predicted spatial patterns for genes with unknown patterns based on the ODE spatial prediction model and 28 prior gene patterns

**Gene**	**Predicted pattern**
‘foxh1.2’	‘v’
‘hnf1b’	‘m’
‘mespb’	‘b’
‘myc’	‘m’
‘myf5’	‘d’
‘myod1’	‘b’
‘sox21’	‘b’
‘sox7’	‘d’

‘d’, ‘v’, ‘b’, ‘u’, ‘m’ stand for dorsal, ventral, both dorsal and ventral, uniform and middle (vegetal) patterns, respectively.

In the Markov spatial prediction model, we supposed the known spatial gene expression patterns were observed at a particular time point *t*2 and they were evolved from a few gene expression patterns at a time point *t*1. It was found that two gene expression patterns (e.g., *bmp4* and *ctnnb1*) at *t*1 could result in 11 (i.e., 39.3%) correctly predicted gene expression patterns at *t*2 using the inferred network from the Markov model for the 28 genes. The correctly predicted genes were: *bmp4, foxh1.1, frzb, gsc, hhex, mix1, msx1, otx2, vegt, ventx1.2, ventx2.2*. Expression patterns of 11 genes (i.e., 39.3%) at *t*2 were correctly predicted when using the same two genes at *t1* and the 36-gene network inferred from the Markov model for the 36 genes. The prediction results are listed in Table 2.

The ODE and Markov spatial prediction models were applied to steady-state patterns and time series patterns, respectively. The models were able to predict the spatial patterns for some of the key genes involved in mesendoderm specification including *vegt, sox17, gata4*, *ventx* and *gsc* when only provided a subnetwork of genes. However, the majority of the predictions between the two models do not overlap and each model predicts a slightly different subset of the core circuitry. Despite only having a single time point of experimental images and using a pre-defined spatial pattern for two genes at an early time point, the Markov model has analogous performance to the ODE model in terms of predicting spatial expression patterns. The correct prediction of different gene subsets of the core mesoderm and endoderm regulatory circuitry by the two models may be suggestive of different underlying spatial dynamics for those genes.

**Figure 9:**
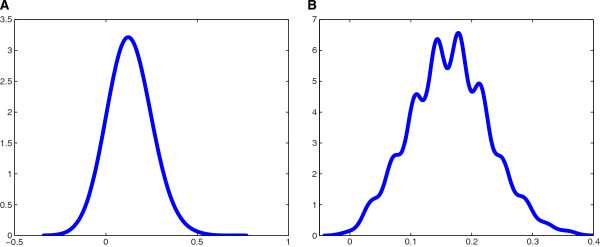
**Estimated probability density distribution of the spatial patterns prediction.** 1000 random networks derived from rearranging the inferred network were used to calculate the fractions of the correctly predicted genes in the 28 genes with prior patterns. **(A)** 1000 random networks were derived from rearranging the inferred 28-gene ODE network. They are mostly about 12% correct (the p-value is 0.007, i.e., there are only 0.7% of the 1000 random networks obtaining not less prior patterns than the inferred ODE network), while the inferred ODE network is 39.3% correct. **(B)** 1000 random networks were derived from rearranging the inferred 28-gene Markov network. They are mostly about 14% correct (the p-value is 0.002, i.e., there are 0.2% of the 1000 random networks obtaining not less prior patterns than the inferred Markov network), while the inferred Markov network is 39.3% correct.

We also compared the two inferred networks with a number of (e.g., 1000) random permutation matrices derived from the inferred networks. We calculated the corresponding fraction of the correctly predicted genes among the 28 genes with prior patterns for each random network derived from rearranging the inferred networks. The probability density estimate is shown in Figure 9. Most random networks derived from rearranging the inferred 28-gene ODE network are about 12% correct (the p-value is 0.007, i.e., there are only 0.7% of the 1000 random networks obtaining not less prior patterns than the inferred ODE network), while the ODE network is 39.3% correct. Similarly, most random networks derived from rearranging the inferred 28-gene Markov network are about 14% correct (the p-value is 0.002, i.e., there are only 0.2% of the 1000 random networks obtaining not less prior patterns than the inferred Markov network), while the Markov network is 39.3% correct.

## Conclusions

There is an increasing need to integrate the approaches that unravel the complicated networks of gene regulatory processes and the works that focus on the spatial-temporal multi-cellular phenomena of pattern formation and morphogenesis. Currently, the network-centric studies produce volumes of regulatory interactions typically with little regard to how these networks specify cellular fate in the context of spatial patterns of gene expression. While development-centric studies focus on small sets of genes, they require labor-intensive approaches, and do not fully embed those genes within the larger regulatory network. Our study represents an initial attempt to integrate these disparate approaches into a single methodology based on biological gene perturbations combined with constraints from spatial modeling. With such an approach, one can make more meaningful predictions for spatial patterns and developmental programs, constrained by the observed complex regulatory networks and in response to changes in gene expression that can be tested experimentally.

We have applied two gene regulatory network inference models with different underlying assumptions to Nanostring experimental data from heterogeneous cell populations from the *Xenopus* embryo. One inference model is an ODE model that assumes steady-state data, and we have previously developed an optimization framework for this model that incorporates prior network information. The other inference model is a Markov model for time series data. We have shown that the Markov model fits within our optimization framework, and extended the model so that prior network information and sparseness constraints can be incorporated directly into the optimization task. We have tested the extended Markov model on simulated network data and showed that existing network information improves performance and can perform well even when some of the existing network information is partially incomplete. In this regard, we have recently obtained ChIP-seq data for *sox17* and RNA-seq after *sox17* MO knockdown [Cho, Blitz and Zorn, unpublished results]. Based on this observation we were able to confirm 4 out of 7 *sox17* connections predicted by the steady-state ODE model, and 3 out of 7 connections predicated by the Markov model. Some of these confirmed interactions include newly predicted connections.

Both inference models were able to recover the core circuitry for controlling dorsal endoderm specification and dorsal mesoderm specification. Differences in the model predictions suggest different dynamics that may be related to the underlying assumptions for each model. For the dorsal endoderm circuitry, the ODE model usually does not differentiate regulatory action of *sox17a* and *sox17b*, while the Markov model sometimes splits their regulatory action and places more connections for *sox17b*. This suggests that even though *sox17a* and *sox17b* are similar in their expression and activity, they may play different roles in their temporal dynamics in their feedback loop with *gata4-6* and *bix1*. A putative hypothesis is that *sox17b* is the primary driver of temporal change for the feedback loop, while *sox17a* stabilizes those changes. For the dorsal mesoderm circuitry, both inference models recovered some core dorsal mesoderm circuit with slightly different gene sets.

Recent experimental results have provided the opportunity to compare our predictions. A T/T2 double knockdown was performed by microinjection of sequence-specific morpholino antisense oligonucleotides, and RNA-seq data of perturbed embryos was obtained at stage 32 [54]. The differential expressions of the selected targets were retrieved from the analyzed datasets for the 36 genes used in this study (see Additional file 7). For comparison purposes, we consider genes with >1.5 fold change to be directly or indirectly regulated by *t* where a negative log fold change indicates positive regulation, while a positive log fold change indicates negative regulation. The results indicate a total of 18 genes regulated by gene *t* (Additional file 7). Among them, *foxa4a, gsc, mespb, myf5, mix1, bix1.2, myod1* (i.e., totally 7 genes) are positively regulated by *t*, and *bmp4, sox21, vegt, ventx2.2, ventx1.2, msx1, wnt11, foxh1.1, nodal6, mixer, nodal3* (i.e., totally 11 genes) are negatively regulated by *t*. Using the forward ODE and Markov models (described in Methods), the forward ODE model predicts the positively regulated genes are *foxa4a, myf5* and *bix1.2*; and predicts the negatively regulated genes are *sox21, vegt, msx1, wnt11, foxh1.1, nodal6* and *mixer.* The forward Markov model predicts the positively regulated genes are *foxa4a, gsc, myf5, mix1* and *myod1*; and predicts the negatively regulated genes are *bmp4, vegt, foxh1.1, nodal6* and *mixer.*

Given the two inferred regulatory networks from the ODE model and the Markov model, we additionally constrained these networks by using them to predict spatial gene expression in the *Xenopus* embryo. Both models were able to predict the spatial patterns for some of the key genes involved in mesoendoderm specification. Interestingly, each model tended to correctly predict a different subset of genes suggesting that those genes are playing different roles in the spatial-temporal dynamics.

The spatial prediction model is dependent upon the provided inferred network for how well it can predict spatial patterns. Similar to the inferred network, the number of prior predicted spatial patterns should not be directly interpreted as the accuracy of the algorithm, because there are numerous reasons why not all of the prior patterns were predicted. A number of (not-inclusive) reasons include 1) incomplete or incorrect connections in the inferred network, 2) incorrect or coarse pattern classification for the biological spatial images, or 3) the non-linear spatiotemporal dynamics are not accurately captured in our 1-dimensional abstract model. However, the fact that a statistically significant portion of the prior patterns is predicted suggests that the spatial prediction algorithm is effectively utilizing the information it is given. Given a more accurate inferred network, the spatial predictions should improve.

One limitation of the spatial prediction with regards to the Markov model is that we did not have access to temporal spatial gene expression images. Instead, we took the approach of assuming initial spatial patterns for a small set of well-studied genes for an earlier time point, then tested the model ability to predict spatial patterns at a later time point. Despite being given only limited initial data, the Markov model was able to correctly predict 39.3% of the gene expression patterns and thus suggesting that the model is accurately capturing some aspects of the temporal dynamics involved in early *Xenopus* development. In the future, *in situ* images of gene expression patterns at multiple embryo stages could improve the prediction capability of the Markov model.

## Methods

### Morpholino knockdown and NanoString analysis of gene expression

Synchronously developing *Xenopus tropicalis* embryos were obtained by *in vitro* fertilization, dejellied in pH 7.6 3% cysteine prepared in 1/9^th^X MMR, and cultured on agarose-coated plates until they reached the 2-4-cell stage [55]. Embryos were microinjected in 1X MMR with translation-blocking morpholino antisense oligonucleotides (MO) that targeted *foxh1* (22.5 ng), *vegt* (22.5 ng), *sox17α/β* (20 ng), or *ctnnb1* (β-catenin) (10 ng) and cultured in 1/9^th^X MMR at 25°C. Sequences of the MOs used in this study were:

•*foxh1* (TCATCCTGAGGCTCCGCCCTCTCTA)

•*vegt* (TGTGTTCCTGACAGCAGTTTCTCAT)

•*ctnnb1* (TTTCAACAGTTTCCAAAGAACCAGG)

•*sox17a* (AGCCACCATCAGGGCTGCTCATGGT)

•*sox17b1/b2* (AGCCACCATCTGGGCTGCTCATGGT)

Total RNA from three biologically independent samples were prepared at the stages of interest using the acid guanidinium thiocyanate phenol chloroform method [56]. Measurements of transcript abundances were performed using the NanoString platform [57]. In brief, RNA sample (total 100 ng) was hybridized to probe sets at 65°C for a minimum of 18 hours. The hybridized probes were recovered using the NanoString Prep Station, and immediately evaluated using the NanoString nCounter. For each reaction 1155 fields of view were counted. Detailed protocols for NanoString transcript counting followed the manufacturer’s instruction manual (http://www.nanostring.com/).

### A steady-state ODE model

We model the gene network inference as the following linear ordinary differential equations (ODEs)

(1.1) $$\frac{dx_{i}^{k}}{\mathrm{dt}}=\sum_{j=1}^{p}W_{\mathrm{ij}}x_{j}^{k},$$

where $x_{i}^{k}$ is the concentration of mRNA for gene *i* measured at the *k* th observation (e.g., time point, sample, wild type expression, knockout expression etc.), $\frac{dx_{i}^{k}}{\mathrm{dt}}$ is the rate of change for the mRNA concentration of gene *i* at the *k* th observation, *p* is the number of genes, *W* is the gene interaction matrix which is to be inferred.

If the ODEs system is close to a steady state, i.e., the change of gene concentrations is very small, then $\frac{dx_{i}^{k}}{\mathrm{dt}}\approx 0$ and as a result $\sum_{j=1}^{p}W_{\mathrm{ij}}x_{j}^{k}\approx 0$. Here we consider how one gene is affected by the other genes, so we can let the diagonal of *W* be -1 s, then $\sum_{\begin{array}{c} j=1\\ j\neq i \end{array}}^{p}W_{\mathrm{ij}}x_{j}^{k}\approx x_{i}^{k}$, which was also used in [58]. Based on our previous work [17], which incorporated two regularization terms, i.e., a sparsity control and the prior network information, we transform the linear steady-state ODE model to the following optimization problem:

(1.2) $$\min_{W}\sum_{k=1}^{n}\sum_{i=1}^{p}{\left(\sum_{\begin{array}{c} j=1\\ j\neq i \end{array}}^{p}W_{\mathrm{ij}}{\bar{x}}_{j}^{k}-x_{i}^{k}\right)}^{2}+\alpha {∥W∥}_{1}+\beta {∥W\circ W^{0}∥}_{1},$$

where *n* is the number of observations, *α* is a positive parameter that enforces the sparsity of the interaction network, ${∥W∥}_{1}=\sum_{i=1}^{p}\sum_{j=1}^{p}\left|W_{\mathrm{ij}}\right|,W^{0}$ is a Boolean network containing existing network information where $W_{\mathrm{ij}}^{0}=0$ indicates a directed interaction from gene *j* to gene *i* and thus is not penalized while $W_{\mathrm{ij}}^{0}=1$ for all the other edges and *β* ≥ 0 indicates the strength of the penalty. ${\bar{x}}^{k}=x^{k}$ if *x*^*k*^ is a wild-type data point. If *x*^*k*^ is a knockout data, we previously put forward one method from a perturbation aspect [17]. Here we suggest another but simple way as follows. Since a knocked out gene does not contribute to the change of other genes’ concentrations, we can let ${\bar{x}}_{j}^{k}=x_{j}^{k}$ for all *j* = 1,…, *p* except ${\bar{x}}_{i}^{k}=0$ if gene *i* is knocked out at a single observation *k*. All these notations are used throughout the paper with the same meanings.

### A Markov model

If time series data is achievable, the finite difference $\frac{x_{i}^{k+1}-x_{i}^{k}}{\mathrm{\Delta t}}$ can be considered to approximate the rate of change of gene $i\left(i.e.,\frac{dx_{i}^{k}}{\mathrm{dt}}\right)$[20], but when time intervals are long (e.g., in hours), this approximation is very inaccurate. [20,27] suggested to employ a linear first order Markov model, which assumed the expression of genes at time *k*, as a linear function of its regulators at the previous time k – 1, i.e., $x_{i}^{k}=\sum_{j=1}^{p}W_{\mathrm{ij}}x_{j}^{k-1}$. In this paper, we combine the idea of linear Markov model and regularization. We first put forward a linear Markov model incorporating network sparsity control and prior network information, which can be shown in the following optimization form:

(1.3) $$\min_{W}\sum_{\mathrm{obs}=1}^{\mathrm{ob}s_{\max}}\sum_{k=1}^{k_{\max}}{∥W{\left({\bar{x}}^{k}\right)}^{\left(\mathrm{obs}\right)}-{\left(x^{\left(k+1\right)}\right)}^{\left(\mathrm{obs}\right)}∥}^{2}+\alpha {∥W∥}_{1}+\beta {∥W\circ W^{0}∥}_{1},$$

where *obs*_max_ represents the number of observations. Here one observation means one sample (e.g., wild-type data or knockdown data) with a complete time series, *k*_max_ represents the number of time points in each observation, and all the other notations have the same meanings as before. In particular, ${\bar{x}}^{k}=x^{k}$ if *x*^*k*^ is a wild-type data point. If gene *i* is knocked down at time *k* in one observation *obs* we can let ${\left({\bar{x}}_{j}^{k}\right)}^{\mathrm{obs}}={\left(x_{j}^{k}\right)}^{\mathrm{obs}}$ for all *j* = 1,…, *p* except *i* and ${\left({\bar{x}}_{i}^{k}\right)}^{\mathrm{obs}}=0$. Notice, here we do not require *j* ≠ *i* as we did in (Eq. 1.2) since for gene *i* the concentration at time $k\left(i.e.,{\bar{x}}_{i}^{k}\right)$ could contribute to that at time $k+1\left(i.e.,x_{i}^{k+1}\right)$.

Previously, we presented an optimization framework to solve a linear gene network inference with steady-state data [17]. We found that the optimization problems (Eq. 1.2) and (Eq. 1.3) happen to be its special cases and they are equivalent to the following optimization problem:

(1.4) $$\begin{array}{ll} \min_{W}\mathrm{tr}\left(W^{T}W\Sigma \right) & -2\mathrm{tr}\left(\mathrm{WU}\right)+\sum_{i=1}^{p}W_{i\cdot }D^{i}W_{i\cdot }^{t}\\ & +\alpha {∥W∥}_{1}+\beta {∥W\circ W^{0}∥}_{1}, \end{array}$$

where *tr* is the trace of a matrix, the matrices Σ and *U* for the linear ODE model are

(1.5) $$\sum =\sum_{k=1}^{n}{\bar{x}}^{k}{\left({\bar{x}}^{k}\right)}^{T},U=\sum_{k=1}^{n}{\bar{x}}^{k}{\left(x^{k}\right)}^{T},$$

the matrices Σ and *U* for the linear Markov model are

(1.6) $$\begin{array}{l} \sum =\sum_{\mathrm{obs}=1}^{\mathrm{ob}s_{\max}}\sum_{k=1}^{k_{\max}}{\left({\bar{x}}^{k}\right)}^{\left(\mathrm{obs}\right)}{\left({\left({\bar{x}}^{k}\right)}^{\left(\mathrm{obs}\right)}\right)}^{T},\\ U=\sum_{\mathrm{obs}=1}^{\mathrm{ob}s_{\max}}\sum_{k=1}^{k_{\max}}{\left({\bar{x}}^{k}\right)}^{\left(\mathrm{obs}\right)}{\left({\left(x^{k+1}\right)}^{\left(\mathrm{obs}\right)}\right)}^{T}, \end{array}$$

*W*_*i*_ denotes the *i*-th row of the matrix *W*, *D*^*i*^ is a *p* × *p* zero matrix for *i* = 1,…, *p*.

In some cases partial gene concentrations are not available, e.g., different gene sets may be chosen in different experiments. Mathematically, suppose in the linear ODE model the concentration of gene *i* at observation *k* is not available $\left(i.e.,x_{i}^{k}=0\right)$, then ${\left(\sum_{j=1,j\neq i}^{p}W_{\mathrm{ij}}{\bar{x}}_{j}^{k}-x_{i}^{k}\right)}^{2}$ need to be deleted from the sum in (Eq. 1.2) because $x_{i}^{k}$ is not predictable. The matrices Σ, *U* and *D*^*i*^ in the optimization problem (Eq. 1.4) should change correspondingly. If *W*_*ii*_ is always set to be 0 and define *I*_*i*_ to be the set of observation indices of gene *i* with zero concentrations, i.e., $I_{i}=\left\{k|k=1,\ldots ,n\right\}\cap \left\{k|x_{i}^{k}=0\right\}$, then $\sum_{i=1}^{p}{\sum_{k=1}^{n}\left(\sum_{\begin{array}{c} j=1\\ j\neq i \end{array}}^{p}W_{\mathrm{ij}}{\bar{x}}_{j}^{k}-x_{i}^{k}\right)}^{2}$ in (Eq. 1.2) should be replaced by ${\sum_{i=1}^{p}\sum_{\begin{array}{c} k=1\\ k\notin I_{i} \end{array}}^{n}\left(\sum_{\begin{array}{c} j=1\\ j\neq i \end{array}}^{p}W_{\mathrm{ij}}{\bar{x}}_{j}^{k}-x_{i}^{k}\right)}^{2}$, which can be transformed as follows:

(1.7) $$\begin{array}{ll} \sum_{i=1}^{p}\sum_{\begin{array}{c} k=1\\ k\notin I_{i} \end{array}}^{n}{\left(\sum_{\begin{array}{c} j=1\\ j\neq i \end{array}}^{p}W_{\mathrm{ij}}{\bar{x}}_{j}^{k}-x_{i}^{k}\right)}^{2} & =\sum_{i=1}^{p}\sum_{\begin{array}{c} k=1\\ k\notin I_{i} \end{array}}^{n}{\left(\sum_{j=1}^{p}W_{\mathrm{ij}}{\bar{x}}_{j}^{k}-x_{i}^{k}\right)}^{2}\\ & =\sum_{i=1}^{p}\sum_{\begin{array}{c} k=1\\ k\notin I_{i} \end{array}}^{n}\left(W_{i\cdot }\bar{x}-x_{i}^{k}\right){\left(W_{i\cdot }{\bar{x}}^{k}-x_{i}^{k}\right)}^{T}\\ & =\sum_{i=1}^{p}\sum_{\begin{array}{c} k=1\\ k\notin I_{i} \end{array}}^{n}W_{i\cdot }{\bar{x}}^{k}{\left({\bar{x}}^{k}\right)}^{T}W_{i\cdot }^{T}\\ & \;-2\sum_{i=1}^{p}\sum_{\begin{array}{c} k=1\\ k\notin I_{i} \end{array}}^{n}W_{i\cdot }{\bar{x}}^{k}x_{i}^{k}+c\\ & =\sum_{i=1}^{p}W_{i\cdot }\left(\sum_{\begin{array}{c} k=1\\ k\notin I_{i} \end{array}}^{n}{\bar{x}}^{k}{\left({\bar{x}}^{k}\right)}^{T}\right)W_{i\cdot }^{T}\\ & \;-2\sum_{i=1}^{p}W_{i\cdot }\sum_{k=1}^{n}{\bar{x}}^{k}x_{i}^{k}+c\\ & =\sum_{i=1}^{p}W_{i\cdot }\left(\sum_{\begin{array}{c} k=1\\ k\notin I_{i} \end{array}}^{n}{\bar{x}}^{k}{\left({\bar{x}}^{k}\right)}^{T}\right)W_{i\cdot }^{T}\\ & \;-2\mathrm{tr}\left(W\sum_{k=1}^{n}{\bar{x}}^{k}{\left(x^{k}\right)}^{T}\right)+c, \end{array}$$

where *c* is a constant independent of *W*. Compared with the form in (Eq. 1.4), it can be found that for the ODE model

(1.8) $$\sum =\mathrm{zero}\;\mathrm{matrix},\;D^{i}=\sum_{\begin{array}{c} k=1\\ k\notin I_{i} \end{array}}^{n}{\bar{x}}^{k}{\left({\bar{x}}^{k}\right)}^{T},\;U=\sum_{k=1}^{n}{\bar{x}}^{k}{\left(x^{k}\right)}^{T}.$$

Similarly, for the Markov model

(1.9) $$\begin{array}{l} \sum =\;\mathrm{zero}\;\mathrm{matrix},\;D^{i}=\sum_{\begin{array}{l} k=1:k_{\max}\\ \mathrm{obs}=1:\;\mathrm{obs}\\ \left(k,\;\mathrm{obs}\right)\notin I_{i} \end{array}}{\left({\bar{x}}^{k}\right)}^{\mathrm{obs}}{\left({\left({\bar{x}}^{k}\right)}^{\mathrm{obs}}\right)}^{T},\\ U=\sum_{\mathrm{obs}=1}^{\mathrm{obs}\_\max}\sum_{k=1}^{k\_\max}{\left({\bar{x}}^{k}\right)}^{\left(\mathrm{obs}\right)}{\left({\left(x^{k+1}\right)}^{\left(\mathrm{obs}\right)}\right)}^{T}, \end{array}$$

where $I_{i}=\left\{\left(p,q\right)|p=2,\ldots ,k_{\max}+1;q=1,\ldots ,\mathrm{ob}s_{\max}\right\}\cap \left\{\left(p,q\right)|{\left(x_{i}^{p}\right)}^{q}=0\right\}$. As a conclusion, the following simplified optimization framework can be used for both the ODE model (Eq. 1.2) and the Markov model (Eq. 1.3) with all kinds of data (e.g., wild-type data, knockdown data or data with partial zeros):

(1.10) $$\begin{array}{cc} \min_{W} & \sum_{i=1}^{p}W_{i\cdot }D^{i}W_{i\cdot }^{T} \end{array}-2\mathrm{tr}\left(\mathrm{WU}\right)+\alpha {∥W∥}_{1}+\beta {∥W\circ W^{0}∥}_{1},$$

where the matrices *D*^*i*^ and *U* are defined in (Eq. 1.8) or (Eq. 1.9).

The optimization problem (Eq. 1.10) can be solved by combining an iterative coordinate descent algorithm for a given pair of parameters (*α*, *β*) and a leave-one-out cross-validation to find the optimal values of (*α*, *β*) which provide the minimal cross-validation error [17,59]. Here ‘leave-one-out’ means to leave one ‘observation’ out, which implies all the time-series data in one observation are left out for the Markov model. We perform an exponential search starting from max  |*U*_*ij*_|, where *U* is defined in (Eq. 1.8) or (Eq. 1.9), and going down. Since the incoming edges for each gene in the gene network can be considered independent from the incoming edges of other genes, we used a separate *α* and *β* for each gene. For each gene and (*α*, *β*), zero initial and an accuracy control of 10^-5^ are used in the optimization procedure.

### Knockout simulations

Here we consider numerical simulations of knocking out genes in a forward ODE model and a forward Markov model (more computational details are provided in Additional file 1).

(1) A Forward ODE Model

We define a forward ODE model as following

(1.11) $$\frac{\mathrm{dx}}{\mathrm{dt}}=\mathrm{Wx}-x,$$

where *W* ∈ *R*^*p × p*^ is the inferred network derived from the ODE model (Eq. 1.2) with zero diagonal elements, *x* ∈ *R*^*p*^ represents a vector of *p* gene expressions.

We use the 4th-order Runge–Kutta method to solve the above ODE. Choose the initial vector as a random vector drawn from the uniform distribution between 0 and 1. We next consider the following persistent complete knockout. Suppose the set of knocked down genes is denoted as K, keep *x*_*i*_ = 0 (*i* ∈ K).

(2) A Forward Markov Model

We define a forward Markov model as following

(1.12) $$x^{k+1}=Wx^{k},$$

where *W* ∈ *R*^*p × p*^ is the inferred network derived from the Markov model (Eq. 1.3), *x*^*k*^ ∈ *R*^*p*^ (*k* = 1,2,…) is the *k*-th iteration vector of *p* gene expressions.

We define the initial vector *x*^1^ as a random vector drawn from the uniform distribution between 0 and 1. We consider the similar knockout as before. The only difference is to add a superscript *k*, i.e., keep $x_{i}^{k}=0$ (*i* ∈ K) for all *k*.

### Spatial prediction model

Given a regulatory network, known spatial expression patterns for genes in the network can be used to predict the unknown spatial patterns for the remaining genes in the network. Examples of typical spatial gene expression patterns in *Xenopus* gastrula stage embryos are shown in Figure 7. For simplicity, we regard the dorsal-ventral axis as a one-dimensional interval, which is partitioned into three regions, i.e., right (dorsal), middle (vegetal) and left (ventral). We took a set of 28 *in situ* images of the 36 total genes being considered in this study, and categorized the gene expression level for the three regions as either low, medium or high within a given embryo. For computational purposes, we assigned arbitrary values to these levels of 0.1 (low), 0.4 (medium) and 1.0 (high) (the effect of varying these values is provided in Additional file 1). The overall spatial expression pattern could then be assigned a category of dorsal (‘d’), ventral (‘v’), both dorsal and ventral (‘b’), vegetal (‘m’) or uniform (‘u’). The expression values across the three regions are (0.1, 0.4, 1.0) for “dorsal” and (1.0, 0.4, 0.1) for “ventral”. The other categories have multiple possible values with “both” having higher dorsal and ventral values than the middle region (e.g. 1.0, 0.1, 1.0), “vegetal” having a higher value in the middle region than the dorsal and ventral regions (e.g. 0.1, 1.0, 0.1), and “uniform” having the same values across all regions (e.g. 0.4, 0.4, 0.4). The categorized spatial gene expression patterns are shown in Table 2.

For steady-state patterns we define the following optimization problem:

(1.13) $$\begin{array}{l} \min_{\left\{x_{i}^{\mathrm{pos}}\right\}}\;\sum_{i=1}^{p}(x_{i}^{\mathrm{pos}}-\sum_{\begin{array}{l} j=1\\ j\neq i \end{array}}^{p}W_{\mathrm{ij}}x_{j}^{\mathrm{pos}}){}^{2}\\ \mathrm{subject}\;\mathrm{to}\;x_{k}^{\mathrm{pos}}=y_{k}^{\mathrm{pos}},\;k\in \mathrm{Index},\\ x_{i}^{\mathrm{pos}}\geq 0,\;i=1,\ldots ,p,\;\mathrm{pos}=1,2,3, \end{array}$$

where *pos* represents the three one-dimensional regions of the embryo, *Index* is the set of genes with known patterns, $y_{k}^{\mathrm{pos}}$ represents the concentration of gene *k* with known patterns at position *pos* (i.e. one of 1, 0.4 and 0.1), and *W* is the inferred network from the steady-state ODE model. This optimization problem (Eq. 1.13) considers the inferred network *W* as fixed, thus directly using the coefficients and gene-to-gene interaction of *W* to define the structure of the spatial prediction model. Some of the $X_{i}^{\mathrm{pos}}$ values are provided as known gene spatial expression patterns, while the remaining $X_{i}^{\mathrm{pos}}$ values are free variables. The resultant values of the free variables are interpreted as spatial predictions for their associated genes. The optimization problem (Eq. 1.13) satisfies the known gene spatial patterns while simultaneously constraining the model with the gene-to-gene interaction topology from the steady-state equations in the ODE model (Eq. 1.1), so we call (Eq. 1.13) ODE spatial prediction model, which is a quadratic programming and can be solved by the MATLAB function ‘lsqlin’.

The ODE spatial prediction model does not contain time information. In the following, we put forward a Markov spatial prediction model for time series data. Suppose spatial gene expressions for a subset of genes are observed or defined at time points *t1* and *t2*. Then we can calculate the spatial gene expressions for other genes at time *t*2. For each region we define the following optimization model:

(1.14) $$\begin{array}{l} \min_{\left\{x_{i}^{\mathrm{pos},2}\right\}}\;\sum_{i=1}^{N}(x_{i}^{\mathrm{pos},2}-\sum_{j=1}^{N}W_{\mathrm{ij}}x_{j}^{\mathrm{pos},1}){}^{2}\\ \mathrm{subject}\;\mathrm{to}\;x_{k}^{\mathrm{pos},1}=y_{k}^{\mathrm{pos},1},\;k\in \mathrm{Index}1,\\ x_{i}^{\mathrm{pos},2}\geq 0,\;i=1,\ldots ,N,\;\mathrm{pos}=1,2,3, \end{array}$$

where *pos* represents the three one-dimensional regions of the embryo, *N* is the number of genes, $y_{k}^{\mathrm{pos},1}$ represents the concentration of gene *k* with known patterns at position *pos* (i.e. one of 1, 0.4 and 0.1) at time *t*1, $x_{k}^{\mathrm{pos},\;1}$ and $x_{k}^{\mathrm{pos},2}$ represent the concentrations of gene *k* at position *pos* at time *t*1 and *t*2, respectively, *W* is the inferred network from the Markov model, *Index1* is the index set of genes observed at time t1. This optimization problem (Eq. 1.14) considers the inferred network *W* as fixed, thus directly using the coefficients and gene-to-gene interaction of *W* to define the structure of the spatial prediction model. Since no information is available for the initial patterns of the other genes (i.e., $x_{k}^{\mathrm{pos},1},k\in \left\{1,\ldots ,N\right\}\backslash \mathrm{Index}1$), when we solved the optimization problem (1.14) we used random initial patterns (for genes *k*, *k* ∈ {1, …, *N*}\*Index*1) for 1000 optimization runs and averaged the values of $x_{k}^{\mathrm{pos},2}$ as the final prediction. Since $\left\{x_{k}^{\mathrm{pos},1}\right\}$ are all defined, this model is actually a direct computation for $\left\{x_{k}^{\mathrm{pos},2}\right\}$ with a nonnegative constraint. We attempted to leave the expression values for the other genes at time *t1* to be variables and have the optimization model predict their values. However this results in an underdetermined model with an infinite set of solutions.

To determine the spatial categorization based upon the results produced by the optimization algorithm, a set of rules was used that compared the values between the three regions. Suppose the left, middle and right regions are denoted as ‘l’, ‘m’ and ‘r’, respectively. One threshold ‘TH’ is used and by default *TH* = 0.5 (the effect of varying the threshold is provided in Additional file 1). ‘abs(x)’ stands for the absolute value of x. Let ‘min’ and ‘max’ represent the minimum value and maximum value in abs(l-m), abs(m-r) and abs(l-r), respectively.

1. Check if $\frac{\min}{\max}\geq \mathrm{TH}$ or *max* < 0.1,

If yes, output ‘Uniform (‘u’)’; otherwise, go to step 2.

2. Check if m < l and m < r and $\frac{\mathrm{abs}\left(l-r\right)}{\mathrm{abs}\left(m-r\right)}\leq 0.5$ and $\frac{\mathrm{abs}\left(l-r\right)}{\mathrm{abs}\left(l-m\right)}\leq 0.5$,

If yes, output ‘Both (‘b’)’; otherwise, go to step 3.

3. Check if m > l and m > r,

If yes, output ‘Vegetal (‘m’)’; otherwise, go to step 4.

4. Check if l > m and l > r,

If yes, output ‘Ventral (‘v’)’; otherwise, go to step 5.

5. Check if r > m and r > l,

If yes, output ‘Dorsal (‘d’)’; otherwise, output ‘Not one of the five patterns’.

## Competing interests

The authors declare that they have no competing interests.

## Authors’ contributions

ZZ and SC coded and performed the computational experiments. WTC and ILB performed the Nanostring experiments. ILB and KWYC conceived of the Nanostring experiments, and participated in their design and coordination. ZZ, SC, XX and QN conceived of the computational methodology, and participated in its design and coordination. ZZ and SC drafted the manuscript. All authors read, modified and approved the final manuscript.

## Supplementary Material

Additional file 1 Contains method description for p-value calculation, the effect of varying algorithmic parameters for determining the spatial gene expression patterns, comparison and sensitivity analysis for the forward ODE and Markov models, and Tables S1, S2, S3 and S4. Click here for file

Additional file 2 **Nanostring probes.** The list of 177 genes with the NanoString probes. Click here for file

Additional file 3 **Nanostring data.** NanoString data from the *ctnnb1* and *sox17* morpholino antisense oligonucleotide (MO) experiments. Click here for file

Additional file 4 **Nanostring data.** NanoString data from the *foxh1* and *vegt* morpholino antisense oligonucleotide (MO) and *cers* perturbation experiments. Click here for file

Additional file 5 **Xenbase image data.** Source for the 28 genes of known spatial expression patterns and links to their pictures from Xenbase. Click here for file

Additional file 6 **Xenbase image data.** Publication source for the spatial expression patterns of the 28 genes. Click here for file

Additional file 7 **Gentsch et al. gene expression data.** Differential gene expression for the 36-gene subset after T/T2 double knockdown. Click here for file
