# Supplementary material for: Inference of the Xenopus tropicalis embryonic regulatory network and spatial gene expression patterns
Source: BMC Syst Biol. 2014 Jan 8;8:3. doi: 10.1186/1752-0509-8-3 (PMC3896677; doi:10.1186/1752-0509-8-3)
Supplement: Additional file 1 — Contains method description for p-value calculation, the effect of varying algorithmic parameters for determining the spatial gene expression patterns, comparison and sensitivity analysis for the forward ODE and Markov models, and Tables S1, S2, S3 and S4. [file 1752-0509-8-3-S1.docx]

**Supporting Information**

**Approximate P-value Calculation for Inferred Networks**

We generate 1000 random networks with 694 and 410 interactions respectively, which are exactly same interaction numbers of the inferred ODE network and Markov network respectively. Note that all the networks mentioned in this paper do not include self-regulations. For each randomly generated network, we look at how many prior connections are included and we plot the estimated probability density distribution as shown in Figure S1. Most random networks with 694 interactions contain about 25 prior interactions (the p-value is 0.006) and most random networks with 410 interactions contain about 15 prior interactions (the p-value is 0). The inferred ODE network and Markov network contain 34 and 32 prior interactions (more than 25 or 15), respectively, while there are only 6 out of 1000 random networks with 694 interactions containing more than 34 interactions (or 0 out of 1000 random networks with 410 interactions containing more than 32 prior interactions).

The p-value is calculated as the division of the number of random networks which contain not less prior interactions than the inferred ODE network (or Markov network) by the number of all the random networks (e.g., 1000 here).

Figure S1. Estimated probability density distribution of 1000 randomly generated networks with 694 (A) and 410 (B) interactions, respectively. (A) Most random networks with 694 interactions contain about 25 prior interactions. (B) Most random networks with 410 interactions contain about 15 prior interactions.

**Varying threshold and quantification level parameters for spatial pattern prediction**

The effect of varying the threshold ‘TH’ is displayed in Figure S2. It shows that the number of correctly predicted patterns is non-decreasing as the threshold ‘TH’ increases. This is in accord with our expectation, because that ‘TH’ reflects the proximity of gene expressions in the three (i.e., left, middle and right) regions and that larger ‘TH’ indicates the three gene expressions are closer to each other, thus the predicted ‘uniform’ pattern should be more likely to be correct.

Figure S2. The effect of varying the threshold ‘TH’ of determining the spatial gene expression patterns. It shows that the number of correctly predicted patterns is non-decreasing as the threshold ‘TH’ increases from 0.01 to 1 with the increment 0.01. (A) The inferred 28-gene ODE network is used in the ODE spatial prediction model. We sequentially chose expression patterns of all possible combinations of 27 genes out of the total 28 genes and used them as constraints to predict the remaining gene expression pattern. (B) The inferred 28-gene Markov network is used in the Markov spatial prediction model.

The number of correctly predicted patterns can increase by varying the default values of low, medium and high levels (i.e., 0.1, 0.4 and 1 respectively). Specifically, three random values generated from the uniform distribution between 0 and 1 are sorted and assigned to ‘low’, ‘medium’ and ‘high’ levels respectively. In addition, we assign a randomly generated value from the uniform distribution between 0 and 1 to the initial uniform pattern setting. Then we repeat the same procedure in the 28-gene Markov spatial prediction model for 1000 times. We see that some assignments can result in 16 (i.e., 57.1%) correctly predicted patterns. In contrast, the maximal number of correctly predicted patterns in the ODE spatial model remains to be 11. One reason is that the Markov model involves multiple random initial patterns, takes the average as the final result and is more flexible.


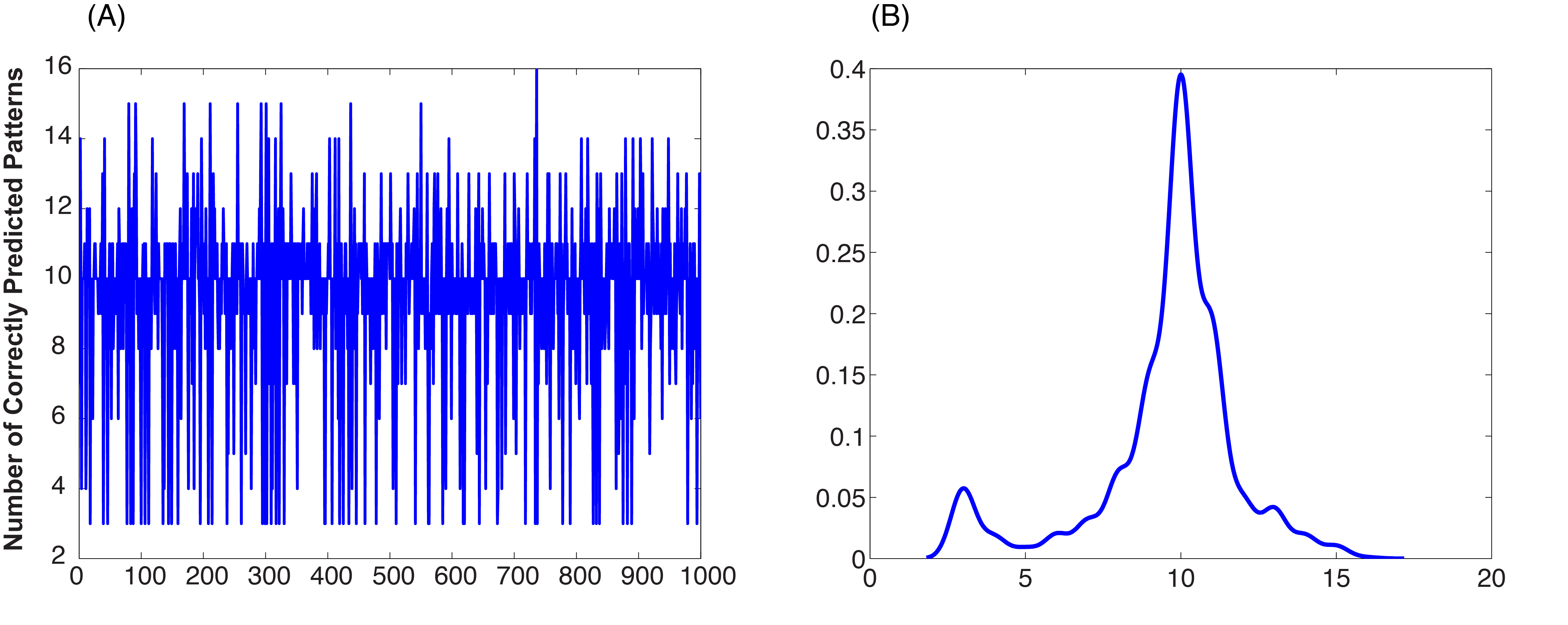


Figure S3. Varying the default values of low, medium and high levels. (A) The number of correctly predicted patterns in the 1000 runs of different assignments for ‘low’, ‘medium’, ‘high’ level values and initial ‘uniform’ pattern setting in the 28-gene Markov spatial prediction model. (B) The estimated probability density distribution of numbers of correctly predicted patterns corresponding to Figure (A).

**Comparison and sensitivity analysis for forward ODE and Markov models**

We observe the change of gene expressions before and after a knockout in the forward ODE model (Eq. 1.11) and forward Markov model (Eq. 1.12). If the gene expression after a knockout decreases, we regard the regulation as ‘activation’. If the gene expression after a knockout increases, we regard the regulation as ‘inhibition’. We solve each model (with or without knockout) for 1000 times with 1000 random initial vectors generated from the uniform distribution between 0 and 1, then take an average over the 1000 solutions as the final result. Taking an average can reduce the bias of random initial values and can be imagined as averaging on cells in the experiments (e.g., one run corresponds to one cell).

For the forward ODE model (Eq. 1.11), we iterate for a relatively long time (e.g., 100 iterations) since the ODE network is derived from the steady-state ODE model (Eq. 1.2). For the forward Markov model (Eq. 1.12), we iterate for a relatively short time (e.g., 5 iterations) since the Markov network is derived from the time-series Markov model (Eq. 1.3) with 4 time points in the *X. tropicalis* data.

There are totally 18 genes regulated by gene *t*. Among them, *foxa4a, gsc, mespb, myf5, mix1, bix1.2, myod1* (i.e., totally 7 genes) are positively regulated (or activated) by *t*, and *bmp4, sox21, vegt, ventx2.2, ventx1.2, msx1, wnt11, foxh1.1, nodal6, mixer, nodal3* (i.e., totally 11 genes) are negatively regulated (or inhibited) by *t*.

We found that 42.86% of the positive regulations, 63.64% of the negative regulations and 55.56% of all the 18 regulations are correctly predicted from the forward ODE model (Eq. 1.11). The correctly predicted positively regulated genes are: *foxa4a, myf5* and *bix1.2*; the correctly predicted negatively regulated genes are: *sox21, vegt, msx1, wnt11, foxh1.1, nodal6* and *mixer*. We also tested 500 iterations and the results were the same.

We found that 71.43% of the positive regulations, 45.45% of the negative regulations and 55.56% of all the 18 regulations are correctly predicted from the forward Markov model (Eq. 1.12). The correctly predicted positively regulated genes are: *foxa4a, gsc, myf5, mix1* and *myod1*; the correctly predicted negatively regulated genes are: *bmp4, vegt, foxh1.1, nodal6* and *mixer*. We also tested 4 iterations, 6 iterations, taking the solutions as the average of the 3^rd^, 4^th^, 5^th^ iterations, average of the 4^th^, 5^th^, 6^th^ iterations, average of the 5^th^, 6^th^, 7^th^ iterations, respectively, in order to observe the solutions around the 5^th^ iteration. The correctly predicted genes are similar and the details are provided below.

Comparing the results of simulations of knocking out *t* in the forward ODE model (Eq. 1.11) and Markov model (Eq. 1.12), we can see that fewer positively regulated genes are correctly predicted from the forward ODE model than the Markov model, but *foxa4a* and *myf5* (i.e., $\frac{2}{3}\approx66.67\%$ of the predictions of the forward ODE model) are correctly predicted by both models. In the meanwhile, fewer negatively regulated genes are correctly predicted from the forward Markov model than the ODE model, but *vegt, foxh1.1, nodal6* and *mixer* (i.e., $\frac{4}{5}=80\%$ of the predictions of the forward Markov model) are correctly predicted by both models.

The predictions of the forward Markov model (Eq. 1.12):

(a) The iteration number is 4:

The correctly predicted positively regulated genes are:

Myf5 mix1 myod1

The correctly predicted negatively regulated genes are:

bmp4 VegT FoxH1.1 nodal6 mixer

The percentage of correctly predicted positive regulations: 42.86%.

The percentage of correctly predicted negative regulations: 45.45%.

The percentage of total correctly predicted regulations: 44.44%.

(b) The iteration number is 6:

The correctly predicted positively regulated genes are:

foxa4a gsc Myf5 myod1

The correctly predicted negatively regulated genes are:

bmp4 VegT FoxH1.1 nodal6 mixer nodal3

The percentage of correctly predicted positive regulations: 57.14%.

The percentage of correctly predicted negative regulations: 54.55%.

The percentage of total correctly predicted regulations: 55.56%.

(c) Take the solutions as the average of the 3^rd^, 4^th^, 5^th^ iterations:

The correctly predicted positively regulated genes are:

foxa4a gsc Myf5 mix1 myod1

The correctly predicted negatively regulated genes are:

bmp4 VegT FoxH1.1 nodal6 mixer

The percentage of correctly predicted positive regulations: 71.43%.

The percentage of correctly predicted negative regulations: 45.45%.

The percentage of total correctly predicted regulations: 55.56%.

(d) Take the solutions as the average of the 4^th^, 5^th^, 6^th^ iterations:

The correctly predicted positively regulated genes are:

foxa4a gsc Myf5 mix1 myod1

The correctly predicted negatively regulated genes are:

bmp4 VegT FoxH1.1 nodal6 mixer nodal3

The percentage of correctly predicted positive regulations: 71.43%.

The percentage of correctly predicted negative regulations: 54.55%.

The percentage of total correctly predicted regulations: 61.11%.

(e) Take the solutions as the average of the 5^th^, 6^th^, 7^th^ iterations:

The correctly predicted positively regulated genes are:

foxa4a gsc Myf5 myod1

The correctly predicted negatively regulated genes are:

VegT FoxH1.1 nodal6 mixer nodal3

The percentage of correctly predicted positive regulations: 57.14%.

The percentage of correctly predicted negative regulations: 45.45%.

The percentage of total correctly predicted regulations: 50%.

**Table S1.** A complete list of the inferred (694) connections from the linear ODE model. The numbers are the inferred interaction strength.

bix1.2 (regulates) foxa2 = 0.164023

bix1.2 (regulates) foxh1.2 = 0.061831

bix1.2 (regulates) frzb = -0.002706

bix1.2 (regulates) gata4 = -0.010302

bix1.2 (regulates) gata6 = 0.058796

bix1.2 (regulates) hhex = 0.019612

bix1.2 (regulates) mix1 = 0.340794

bix1.2 (regulates) mixer = 0.193595

bix1.2 (regulates) msx1 = 0.067602

bix1.2 (regulates) myc = -0.091446

bix1.2 (regulates) nodal3 = -0.075249

bix1.2 (regulates) nodal6 = 0.260156

bix1.2 (regulates) otx2 = -0.010500

bix1.2 (regulates) sox17a = 0.066422

bix1.2 (regulates) sox17b.1 = 0.040407

bix1.2 (regulates) sox21 = -0.107034

bix1.2 (regulates) vegt = 0.375003

bix1.2 (regulates) ventx2.2 = 0.151398

bix1.2 (regulates) wnt11 = -0.025785

bmp4 (regulates) bix1.2 = -0.087593

bmp4 (regulates) ctnnb1 = 0.190153

bmp4 (regulates) foxa1 = 0.105932

bmp4 (regulates) foxa2 = 0.027319

bmp4 (regulates) foxh1 = -0.233011

bmp4 (regulates) foxh1.2 = 0.278139

bmp4 (regulates) gata4 = -0.002827

bmp4 (regulates) hhex = -0.119742

bmp4 (regulates) hnf1b = -0.021790

bmp4 (regulates) mespb = 0.267359

bmp4 (regulates) mix1 = 0.181386

bmp4 (regulates) mixer = 0.464423

bmp4 (regulates) msx1 = 0.332434

bmp4 (regulates) myc = -0.259812

bmp4 (regulates) otx2 = 0.279398

bmp4 (regulates) sox7 = 0.030521

bmp4 (regulates) t = 0.448901

bmp4 (regulates) vegt = -0.230930

bmp4 (regulates) xbp1 = -0.530740

ctnnb1 (regulates) bix1.2 = 0.161377

ctnnb1 (regulates) foxa1 = -0.038805

ctnnb1 (regulates) foxh1 = 0.046881

ctnnb1 (regulates) foxh1.2 = -0.024960

ctnnb1 (regulates) gata6 = -0.019900

ctnnb1 (regulates) gsc = 0.376687

ctnnb1 (regulates) hhex = -0.122092

ctnnb1 (regulates) mix1 = 0.049443

ctnnb1 (regulates) mixer = 0.201559

ctnnb1 (regulates) msx1 = 0.074475

ctnnb1 (regulates) myc = -0.025609

ctnnb1 (regulates) myf5 = 0.003485

ctnnb1 (regulates) nodal3 = 0.001247

ctnnb1 (regulates) nodal6 = 0.098799

ctnnb1 (regulates) otx2 = -0.035986

ctnnb1 (regulates) sox7 = 0.045776

ctnnb1 (regulates) ventx1.2 = -0.058670

ctnnb1 (regulates) xbp1 = -0.026263

foxa1 (regulates) bmp4 = 0.106109

foxa1 (regulates) foxa2 = 0.402160

foxa1 (regulates) foxa4a = 0.022826

foxa1 (regulates) frzb = -0.434400

foxa1 (regulates) gata4 = 0.239598

foxa1 (regulates) gata6 = -0.261896

foxa1 (regulates) hnf1b = 0.496143

foxa1 (regulates) mespb = -0.126738

foxa1 (regulates) mix1 = -0.140401

foxa1 (regulates) mixer = 0.225353

foxa1 (regulates) msx1 = 0.322750

foxa1 (regulates) myc = 0.144341

foxa1 (regulates) nodal6 = 0.034858

foxa1 (regulates) sox17a = -0.424305

foxa1 (regulates) sox17b.1 = -0.147465

foxa1 (regulates) sox21 = 0.027650

foxa1 (regulates) t = 0.099647

foxa1 (regulates) vegt = -0.032810

foxa1 (regulates) ventx1.2 = -0.002322

foxa1 (regulates) ventx2.2 = -0.033703

foxa1 (regulates) wnt11 = 0.578998

foxa1 (regulates) xbp1 = -0.031117

foxa2 (regulates) foxa1 = 0.376240

foxa2 (regulates) gata4 = 0.092102

foxa2 (regulates) gata6 = 0.009430

foxa2 (regulates) gsc = -0.149221

foxa2 (regulates) hhex = -0.216859

foxa2 (regulates) mix1 = -0.216230

foxa2 (regulates) mixer = -0.129638

foxa2 (regulates) msx1 = -0.052147

foxa2 (regulates) myc = 0.065944

foxa2 (regulates) myod1 = 0.058199

foxa2 (regulates) nodal3 = -0.122575

foxa2 (regulates) sox17a = 0.000150

foxa2 (regulates) sox21 = 0.222182

foxa2 (regulates) t = 0.026452

foxa2 (regulates) vegt = -0.238609

foxa2 (regulates) ventx1.2 = -0.020194

foxa2 (regulates) ventx2.2 = -0.044810

foxa2 (regulates) xbp1 = -0.116532

foxa4a (regulates) foxa1 = 0.006855

foxa4a (regulates) foxa2 = 0.178507

foxa4a (regulates) frzb = -0.100882

foxa4a (regulates) gata4 = 0.014494

foxa4a (regulates) gata6 = -0.001574

foxa4a (regulates) gsc = 0.431586

foxa4a (regulates) hhex = -0.048427

foxa4a (regulates) lhx1 = 0.026596

foxa4a (regulates) mespb = 0.000802

foxa4a (regulates) mix1 = 0.339413

foxa4a (regulates) mixer = 0.340190

foxa4a (regulates) msx1 = -0.037654

foxa4a (regulates) myc = -0.050334

foxa4a (regulates) otx2 = 0.276489

foxa4a (regulates) sox17a = -0.072142

foxa4a (regulates) t = 0.116011

foxa4a (regulates) vegt = 0.345626

foxa4a (regulates) wnt11 = 0.013344

foxa4a (regulates) xbp1 = 0.224796

foxh1 (regulates) bix1.2 = 0.154096

foxh1 (regulates) ctnnb1 = 0.205038

foxh1 (regulates) foxa1 = -0.023895

foxh1 (regulates) foxa2 = 0.004520

foxh1 (regulates) foxa4a = -0.109273

foxh1 (regulates) frzb = -0.235843

foxh1 (regulates) gata4 = -0.002223

foxh1 (regulates) hhex = 0.050603

foxh1 (regulates) lhx1 = 0.230744

foxh1 (regulates) mespb = -0.114559

foxh1 (regulates) mix1 = 0.089718

foxh1 (regulates) mixer = 0.033841

foxh1 (regulates) msx1 = -0.140177

foxh1 (regulates) myc = 0.322508

foxh1 (regulates) myf5 = -0.014634

foxh1 (regulates) nodal6 = 0.074321

foxh1 (regulates) otx2 = -0.388361

foxh1 (regulates) sox17a = -0.112330

foxh1 (regulates) sox17b.1 = -0.048751

foxh1 (regulates) sox7 = 0.795131

foxh1 (regulates) ventx1.2 = -0.074732

foxh1 (regulates) wnt11 = -0.034175

foxh1 (regulates) xbp1 = 0.217821

foxh1.2 (regulates) bix1.2 = 0.092066

foxh1.2 (regulates) ctnnb1 = 0.348545

foxh1.2 (regulates) frzb = -0.597167

foxh1.2 (regulates) gata4 = -0.090102

foxh1.2 (regulates) gata5 = -0.012691

foxh1.2 (regulates) gata6 = -0.097257

foxh1.2 (regulates) gsc = 0.080227

foxh1.2 (regulates) hhex = -0.081512

foxh1.2 (regulates) lhx1 = 0.292344

foxh1.2 (regulates) mespb = 0.211016

foxh1.2 (regulates) mix1 = 0.608858

foxh1.2 (regulates) mixer = 0.584501

foxh1.2 (regulates) myc = 0.197784

foxh1.2 (regulates) myf5 = -0.089869

foxh1.2 (regulates) nodal6 = -0.004719

foxh1.2 (regulates) otx2 = -0.709050

foxh1.2 (regulates) sox17b.1 = 0.030228

foxh1.2 (regulates) sox21 = 0.300873

foxh1.2 (regulates) sox7 = 0.118999

foxh1.2 (regulates) vegt = 0.010897

foxh1.2 (regulates) ventx2.2 = 0.122517

foxh1.2 (regulates) wnt11 = 0.085340

foxh1.2 (regulates) xbp1 = 0.227302

frzb (regulates) bix1.2 = -0.166256

frzb (regulates) ctnnb1 = 0.035337

frzb (regulates) foxa1 = -0.026116

frzb (regulates) foxa2 = 0.131744

frzb (regulates) foxh1.2 = -0.165864

frzb (regulates) gata4 = 0.135228

frzb (regulates) gata6 = 0.048036

frzb (regulates) hhex = 0.124125

frzb (regulates) lhx1 = -0.010955

frzb (regulates) mix1 = 0.199033

frzb (regulates) mixer = 0.084286

frzb (regulates) msx1 = -0.027918

frzb (regulates) myc = -0.039628

frzb (regulates) myf5 = 0.195630

frzb (regulates) myod1 = -0.087233

frzb (regulates) otx2 = -0.213874

frzb (regulates) sox17a = 0.337948

frzb (regulates) sox17b.1 = 0.216350

frzb (regulates) sox21 = 0.382552

frzb (regulates) t = 0.364698

frzb (regulates) vegt = 0.202316

frzb (regulates) ventx2.2 = -0.007068

frzb (regulates) wnt11 = 0.059704

frzb (regulates) xbp1 = 0.311138

gata4 (regulates) foxa2 = 0.480960

gata4 (regulates) frzb = 0.432019

gata4 (regulates) gata6 = 0.587999

gata4 (regulates) hnf1b = 0.333225

gata4 (regulates) lhx1 = 0.883983

gata4 (regulates) mix1 = 0.506934

gata4 (regulates) mixer = 0.027819

gata4 (regulates) msx1 = -0.380345

gata4 (regulates) myc = 0.088289

gata4 (regulates) ventx1.2 = -0.045461

gata4 (regulates) xbp1 = -0.582917

gata5 (regulates) bmp4 = 0.065414

gata5 (regulates) foxa1 = -0.058167

gata5 (regulates) foxa2 = -0.056513

gata5 (regulates) foxh1 = 0.125406

gata5 (regulates) foxh1.2 = -0.208501

gata5 (regulates) gata4 = 0.019574

gata5 (regulates) gata6 = 0.040754

gata5 (regulates) hhex = 0.043655

gata5 (regulates) hnf1b = 0.015175

gata5 (regulates) lhx1 = 0.152380

gata5 (regulates) mix1 = 0.007859

gata5 (regulates) mixer = -0.179744

gata5 (regulates) msx1 = -0.001239

gata5 (regulates) myc = 0.123554

gata5 (regulates) nodal3 = 0.014155

gata5 (regulates) otx2 = -0.356822

gata5 (regulates) sox17b.1 = 0.182297

gata5 (regulates) sox21 = -0.329397

gata5 (regulates) vegt = -0.097158

gata5 (regulates) ventx1.2 = 0.074011

gata5 (regulates) ventx2.2 = 0.119035

gata5 (regulates) wnt11 = 0.118777

gata5 (regulates) xbp1 = 0.267989

gata6 (regulates) foxa1 = -0.265555

gata6 (regulates) foxa2 = 0.094000

gata6 (regulates) foxh1 = -0.105946

gata6 (regulates) foxh1.2 = -0.112507

gata6 (regulates) gata4 = 0.503049

gata6 (regulates) gata5 = 0.586705

gata6 (regulates) hnf1b = 0.434985

gata6 (regulates) lhx1 = 0.070330

gata6 (regulates) mespb = 0.484515

gata6 (regulates) mixer = 0.655544

gata6 (regulates) msx1 = 0.508030

gata6 (regulates) myc = 0.476473

gata6 (regulates) sox17a = 0.298129

gata6 (regulates) sox21 = -0.239535

gata6 (regulates) ventx1.2 = 0.078118

gata6 (regulates) wnt11 = -0.201495

gata6 (regulates) xbp1 = -0.614377

gsc (regulates) bix1.2 = 0.027058

gsc (regulates) ctnnb1 = 0.016614

gsc (regulates) foxa2 = -0.136106

gsc (regulates) foxa4a = 0.282473

gsc (regulates) gata4 = -0.075176

gsc (regulates) gata6 = -0.032216

gsc (regulates) hhex = 0.314072

gsc (regulates) hnf1b = -0.056191

gsc (regulates) lhx1 = 0.038772

gsc (regulates) mespb = 0.099991

gsc (regulates) mix1 = -0.156232

gsc (regulates) mixer = -0.289830

gsc (regulates) myc = 0.125922

gsc (regulates) myf5 = -0.131764

gsc (regulates) nodal3 = 0.198266

gsc (regulates) nodal6 = -0.017238

gsc (regulates) sox17b.1 = 0.125188

gsc (regulates) sox21 = 0.116942

gsc (regulates) sox7 = -0.085488

gsc (regulates) vegt = -0.004852

gsc (regulates) ventx1.2 = -0.068662

gsc (regulates) ventx2.2 = -0.013749

gsc (regulates) xbp1 = -0.273191

hhex (regulates) foxa2 = -0.123169

hhex (regulates) foxh1.2 = -0.002815

hhex (regulates) frzb = 0.224822

hhex (regulates) gata4 = 0.148265

hhex (regulates) gata6 = -0.001056

hhex (regulates) gsc = 0.759054

hhex (regulates) lhx1 = 0.262870

hhex (regulates) mix1 = 0.089649

hhex (regulates) mixer = 0.559420

hhex (regulates) msx1 = 0.167078

hhex (regulates) otx2 = 0.584932

hhex (regulates) vegt = -0.058097

hhex (regulates) wnt11 = 0.628100

hhex (regulates) xbp1 = -0.137024

hnf1b (regulates) foxa1 = 0.429924

hnf1b (regulates) foxh1 = -0.005228

hnf1b (regulates) foxh1.2 = -0.159123

hnf1b (regulates) gata4 = 0.085921

hnf1b (regulates) gata6 = 0.150114

hnf1b (regulates) gsc = -0.029063

hnf1b (regulates) lhx1 = -0.089716

hnf1b (regulates) mespb = -0.086644

hnf1b (regulates) mix1 = 0.161947

hnf1b (regulates) mixer = 0.085867

hnf1b (regulates) msx1 = -0.054815

hnf1b (regulates) myc = -0.158219

hnf1b (regulates) myf5 = 0.041031

hnf1b (regulates) otx2 = -0.135051

hnf1b (regulates) sox21 = 0.239495

hnf1b (regulates) vegt = -0.048889

hnf1b (regulates) ventx1.2 = 0.003905

hnf1b (regulates) xbp1 = 0.320794

lhx1 (regulates) ctnnb1 = 0.214353

lhx1 (regulates) foxh1.2 = 0.096593

lhx1 (regulates) frzb = 0.044591

lhx1 (regulates) gata4 = 0.202168

lhx1 (regulates) gata6 = 0.001045

lhx1 (regulates) hhex = 0.129632

lhx1 (regulates) mespb = 0.058479

lhx1 (regulates) mix1 = -0.233049

lhx1 (regulates) mixer = -0.049827

lhx1 (regulates) msx1 = -0.000869

lhx1 (regulates) myc = -0.402044

lhx1 (regulates) myf5 = 0.288287

lhx1 (regulates) nodal3 = 0.095052

lhx1 (regulates) nodal6 = -0.079746

lhx1 (regulates) otx2 = 0.627047

lhx1 (regulates) sox17b.1 = 0.013584

lhx1 (regulates) vegt = 0.139305

lhx1 (regulates) ventx1.2 = -0.115107

lhx1 (regulates) ventx2.2 = 0.175427

lhx1 (regulates) wnt11 = -0.192952

lhx1 (regulates) xbp1 = 0.515581

mespb (regulates) bix1.2 = -0.048918

mespb (regulates) bmp4 = 0.081841

mespb (regulates) foxh1 = -0.004418

mespb (regulates) foxh1.2 = 0.170586

mespb (regulates) gata4 = -0.022949

mespb (regulates) gata6 = 0.190807

mespb (regulates) gsc = 0.192684

mespb (regulates) hhex = -0.045730

mespb (regulates) lhx1 = 0.294995

mespb (regulates) mix1 = 0.000058

mespb (regulates) mixer = -0.215022

mespb (regulates) msx1 = 0.021408

mespb (regulates) myc = 0.170495

mespb (regulates) myf5 = 0.271051

mespb (regulates) nodal3 = 0.064827

mespb (regulates) sox17a = 0.125491

mespb (regulates) sox17b.1 = 0.057468

mespb (regulates) ventx2.2 = -0.074207

mespb (regulates) wnt11 = -0.065544

mespb (regulates) xbp1 = -0.057508

mix1 (regulates) bix1.2 = 0.427771

mix1 (regulates) foxa2 = -0.213670

mix1 (regulates) foxa4a = 0.270220

mix1 (regulates) foxh1 = -0.199521

mix1 (regulates) foxh1.2 = 0.755526

mix1 (regulates) gata4 = 0.237074

mix1 (regulates) hnf1b = 0.180884

mix1 (regulates) lhx1 = -0.365781

mix1 (regulates) mixer = -0.742366

mix1 (regulates) msx1 = -0.087421

mix1 (regulates) myc = -0.138085

mix1 (regulates) nodal3 = -0.043397

mix1 (regulates) otx2 = 0.947583

mix1 (regulates) sox21 = -0.184241

mix1 (regulates) t = 0.046587

mix1 (regulates) wnt11 = 0.007592

mix1 (regulates) xbp1 = -0.201905

mixer (regulates) foxa1 = 0.114003

mixer (regulates) foxa4a = 0.119703

mixer (regulates) foxh1.2 = 0.299555

mixer (regulates) frzb = 0.135578

mixer (regulates) gata4 = 0.006739

mixer (regulates) gata6 = 0.158917

mixer (regulates) hhex = 0.261237

mixer (regulates) hnf1b = 0.056018

mixer (regulates) lhx1 = 0.060955

mixer (regulates) mix1 = -0.388472

mixer (regulates) msx1 = -0.298701

mixer (regulates) myc = 0.061734

mixer (regulates) myod1 = -0.148742

mixer (regulates) nodal3 = 0.097707

mixer (regulates) sox17b.1 = 0.270158

mixer (regulates) sox21 = -0.536751

mixer (regulates) t = 0.052291

mixer (regulates) vegt = 0.098573

mixer (regulates) ventx1.2 = 0.121207

mixer (regulates) wnt11 = -0.212472

mixer (regulates) xbp1 = 0.133144

msx1 (regulates) bmp4 = 0.094348

msx1 (regulates) foxa1 = 0.442319

msx1 (regulates) foxh1 = -0.186680

msx1 (regulates) frzb = -0.077941

msx1 (regulates) gata4 = -0.254400

msx1 (regulates) gata6 = 0.462885

msx1 (regulates) gsc = 0.104129

msx1 (regulates) mespb = 0.371275

msx1 (regulates) mix1 = -0.138953

msx1 (regulates) mixer = -0.886503

msx1 (regulates) myc = 0.192578

msx1 (regulates) t = 0.063407

msx1 (regulates) ventx1.2 = 0.108822

msx1 (regulates) ventx2.2 = -0.015213

msx1 (regulates) wnt11 = -0.540916

msx1 (regulates) xbp1 = 0.341832

myc (regulates) bmp4 = 0.174318

myc (regulates) foxa1 = 0.054206

myc (regulates) foxh1 = 0.601613

myc (regulates) foxh1.2 = 0.019421

myc (regulates) gata4 = 0.006608

myc (regulates) gata6 = 0.239301

myc (regulates) gsc = 0.224900

myc (regulates) lhx1 = -0.492946

myc (regulates) mix1 = -0.040266

myc (regulates) mixer = 0.214245

myc (regulates) msx1 = 0.196986

myc (regulates) otx2 = 0.666353

myc (regulates) sox17a = 0.179173

myc (regulates) sox7 = 0.051105

myc (regulates) vegt = 0.488514

myc (regulates) ventx2.2 = 0.329936

myc (regulates) wnt11 = 0.627756

myc (regulates) xbp1 = 0.450940

myf5 (regulates) foxa1 = -0.014993

myf5 (regulates) foxa2 = -0.028360

myf5 (regulates) foxa4a = 0.033046

myf5 (regulates) foxh1.2 = 0.009118

myf5 (regulates) frzb = 0.036876

myf5 (regulates) gata4 = -0.076701

myf5 (regulates) gata6 = 0.081328

myf5 (regulates) gsc = -0.279022

myf5 (regulates) hnf1b = 0.087022

myf5 (regulates) lhx1 = 0.150422

myf5 (regulates) mespb = 0.323719

myf5 (regulates) mix1 = -0.142529

myf5 (regulates) mixer = -0.193895

myf5 (regulates) msx1 = -0.098648

myf5 (regulates) myc = -0.038240

myf5 (regulates) myod1 = 0.641324

myf5 (regulates) otx2 = 0.197326

myf5 (regulates) sox17a = 0.082487

myf5 (regulates) sox21 = 0.083918

myf5 (regulates) sox7 = -0.013392

myf5 (regulates) vegt = 0.094964

myf5 (regulates) ventx1.2 = -0.002582

myf5 (regulates) ventx2.2 = 0.005607

myf5 (regulates) wnt11 = 0.048804

myf5 (regulates) xbp1 = -0.139071

myod1 (regulates) foxa2 = 0.083048

myod1 (regulates) foxa4a = 0.126286

myod1 (regulates) foxh1 = -0.085635

myod1 (regulates) foxh1.2 = 0.040606

myod1 (regulates) gata4 = -0.070816

myod1 (regulates) gata6 = 0.043685

myod1 (regulates) hhex = 0.010678

myod1 (regulates) hnf1b = 0.045537

myod1 (regulates) mix1 = 0.008210

myod1 (regulates) mixer = -0.082972

myod1 (regulates) msx1 = -0.000719

myod1 (regulates) myc = 0.008284

myod1 (regulates) myf5 = 0.536086

myod1 (regulates) sox17a = 0.225666

myod1 (regulates) sox17b.1 = 0.139594

myod1 (regulates) sox7 = 0.020406

myod1 (regulates) vegt = -0.275942

myod1 (regulates) wnt11 = 0.465232

nodal3 (regulates) foxa2 = -0.002367

nodal3 (regulates) gata6 = -0.005587

nodal3 (regulates) gsc = 0.040444

nodal3 (regulates) hnf1b = 0.032009

nodal3 (regulates) lhx1 = -0.014852

nodal3 (regulates) mix1 = -0.035085

nodal3 (regulates) mixer = 0.119201

nodal3 (regulates) msx1 = -0.051877

nodal3 (regulates) myc = -0.062237

nodal3 (regulates) nodal6 = 0.660605

nodal3 (regulates) otx2 = -0.113862

nodal3 (regulates) sox17a = -0.003298

nodal3 (regulates) sox21 = 0.164695

nodal3 (regulates) sox7 = 0.044202

nodal3 (regulates) vegt = -0.240390

nodal3 (regulates) ventx1.2 = 0.041354

nodal3 (regulates) ventx2.2 = -0.013414

nodal3 (regulates) xbp1 = -0.203988

nodal6 (regulates) bix1.2 = 0.112497

nodal6 (regulates) foxa2 = -0.001350

nodal6 (regulates) foxh1.2 = -0.095034

nodal6 (regulates) gata4 = 0.039720

nodal6 (regulates) hhex = -0.007588

nodal6 (regulates) hnf1b = 0.000404

nodal6 (regulates) lhx1 = -0.008138

nodal6 (regulates) mix1 = -0.036889

nodal6 (regulates) mixer = -0.236219

nodal6 (regulates) msx1 = -0.047682

nodal6 (regulates) myc = 0.099613

nodal6 (regulates) nodal3 = 0.854371

nodal6 (regulates) sox21 = -0.107501

nodal6 (regulates) ventx2.2 = -0.080864

nodal6 (regulates) xbp1 = 0.436480

otx2 (regulates) foxa1 = -0.034426

otx2 (regulates) foxa2 = 0.007306

otx2 (regulates) foxa4a = 0.105370

otx2 (regulates) foxh1 = -0.029544

otx2 (regulates) foxh1.2 = -0.148353

otx2 (regulates) gata6 = -0.038917

otx2 (regulates) gsc = 0.018829

otx2 (regulates) hhex = 0.098183

otx2 (regulates) hnf1b = -0.108794

otx2 (regulates) lhx1 = 0.120225

otx2 (regulates) mix1 = 0.141464

otx2 (regulates) mixer = 0.114536

otx2 (regulates) myc = 0.053019

otx2 (regulates) myod1 = 0.028520

otx2 (regulates) nodal3 = -0.141333

otx2 (regulates) t = -0.005942

otx2 (regulates) vegt = -0.065271

otx2 (regulates) ventx1.2 = -0.051133

otx2 (regulates) ventx2.2 = 0.045021

otx2 (regulates) wnt11 = -0.087719

otx2 (regulates) xbp1 = 0.248805

sox17a (regulates) bix1.2 = -0.092674

sox17a (regulates) foxa1 = 1.325990

sox17a (regulates) foxa2 = -1.038929

sox17a (regulates) foxa4a = 1.110554

sox17a (regulates) gata4 = -0.497917

sox17a (regulates) gata5 = -1.057395

sox17a (regulates) gata6 = 0.479541

sox17a (regulates) hnf1b = 1.413190

sox17a (regulates) mix1 = -0.142771

sox17a (regulates) mixer = -1.292094

sox17a (regulates) myc = -0.108761

sox17b.1 (regulates) bix1.2 = -0.148399

sox17b.1 (regulates) foxa1 = -0.287426

sox17b.1 (regulates) foxa2 = 0.332471

sox17b.1 (regulates) foxh1 = -0.000377

sox17b.1 (regulates) foxh1.2 = 0.012552

sox17b.1 (regulates) frzb = -0.174458

sox17b.1 (regulates) gata4 = 0.191002

sox17b.1 (regulates) gata5 = 0.224070

sox17b.1 (regulates) gata6 = 0.017174

sox17b.1 (regulates) hhex = -0.138195

sox17b.1 (regulates) hnf1b = -0.363048

sox17b.1 (regulates) mespb = -0.115609

sox17b.1 (regulates) mixer = 0.274068

sox17b.1 (regulates) msx1 = -0.139884

sox17b.1 (regulates) nodal6 = -0.027556

sox17b.1 (regulates) sox17a = -0.063424

sox17b.1 (regulates) sox21 = 0.311895

sox17b.1 (regulates) sox7 = 0.017284

sox17b.1 (regulates) vegt = 0.000336

sox17b.1 (regulates) xbp1 = 0.232981

sox21 (regulates) ctnnb1 = 0.091565

sox21 (regulates) foxa2 = 0.001816

sox21 (regulates) foxh1 = 0.009876

sox21 (regulates) foxh1.2 = 0.171016

sox21 (regulates) frzb = 0.425755

sox21 (regulates) gata4 = 0.105743

sox21 (regulates) gata5 = -0.062582

sox21 (regulates) gata6 = -0.068396

sox21 (regulates) gsc = 0.144728

sox21 (regulates) hnf1b = 0.014589

sox21 (regulates) lhx1 = -0.092684

sox21 (regulates) mespb = -0.072071

sox21 (regulates) mix1 = -0.197317

sox21 (regulates) mixer = -0.243184

sox21 (regulates) myc = 0.002139

sox21 (regulates) myf5 = 0.152112

sox21 (regulates) nodal3 = 0.159314

sox21 (regulates) nodal6 = -0.097943

sox21 (regulates) otx2 = 0.075366

sox21 (regulates) sox7 = 0.078469

sox21 (regulates) t = -0.097878

sox21 (regulates) vegt = 0.022548

sox21 (regulates) ventx2.2 = 0.228372

sox21 (regulates) wnt11 = -0.061877

sox21 (regulates) xbp1 = 0.292044

sox7 (regulates) ctnnb1 = 0.440086

sox7 (regulates) foxh1 = 0.713959

sox7 (regulates) foxh1.2 = 0.036518

sox7 (regulates) gata4 = -0.074270

sox7 (regulates) gata6 = 0.008366

sox7 (regulates) gsc = -0.203540

sox7 (regulates) lhx1 = 0.000214

sox7 (regulates) mix1 = -0.137759

sox7 (regulates) mixer = -0.209206

sox7 (regulates) otx2 = 0.280242

sox7 (regulates) sox21 = 0.200414

sox7 (regulates) t = -0.106451

sox7 (regulates) vegt = 0.025796

sox7 (regulates) ventx2.2 = 0.062157

sox7 (regulates) xbp1 = -0.439569

t (regulates) foxa1 = 0.037333

t (regulates) foxa2 = 0.056392

t (regulates) foxa4a = 0.065186

t (regulates) foxh1 = -0.009325

t (regulates) foxh1.2 = -0.460588

t (regulates) frzb = 0.050053

t (regulates) gata4 = -0.020048

t (regulates) gata6 = -0.183754

t (regulates) gsc = -0.079512

t (regulates) hhex = 0.171372

t (regulates) hnf1b = -0.548338

t (regulates) lhx1 = -0.289532

t (regulates) mespb = 0.012701

t (regulates) mix1 = 0.054516

t (regulates) mixer = 0.155586

t (regulates) msx1 = 0.100103

t (regulates) myc = -0.173712

t (regulates) nodal6 = 0.042963

t (regulates) otx2 = -0.319650

t (regulates) sox7 = -0.070905

t (regulates) vegt = 0.055782

t (regulates) ventx1.2 = 0.265220

t (regulates) xbp1 = 0.159254

vegt (regulates) bix1.2 = 0.232089

vegt (regulates) foxa2 = -0.241530

vegt (regulates) foxa4a = 0.129459

vegt (regulates) foxh1 = 0.102777

vegt (regulates) foxh1.2 = -0.085924

vegt (regulates) frzb = 0.491150

vegt (regulates) gata4 = 0.079859

vegt (regulates) gata6 = -0.226255

vegt (regulates) gsc = -0.110843

vegt (regulates) mix1 = 0.031780

vegt (regulates) mixer = 0.311390

vegt (regulates) msx1 = 0.159868

vegt (regulates) myc = 0.101695

vegt (regulates) myod1 = -0.014494

vegt (regulates) nodal3 = -0.263635

vegt (regulates) nodal6 = 0.002399

vegt (regulates) otx2 = -0.504257

vegt (regulates) sox17b.1 = 0.070829

vegt (regulates) sox21 = 0.029800

vegt (regulates) sox7 = 0.119522

vegt (regulates) ventx1.2 = 0.049980

vegt (regulates) ventx2.2 = 0.001356

vegt (regulates) xbp1 = -0.237359

ventx1.2 (regulates) bmp4 = 0.166616

ventx1.2 (regulates) foxa1 = -0.331270

ventx1.2 (regulates) foxh1 = -0.132099

ventx1.2 (regulates) gata4 = -0.254055

ventx1.2 (regulates) gata5 = 0.560261

ventx1.2 (regulates) gata6 = 0.153860

ventx1.2 (regulates) gsc = -0.467745

ventx1.2 (regulates) hhex = 0.022253

ventx1.2 (regulates) mix1 = 0.253318

ventx1.2 (regulates) mixer = 0.438546

ventx1.2 (regulates) myf5 = -0.335068

ventx1.2 (regulates) otx2 = -0.743949

ventx1.2 (regulates) t = 0.205697

ventx1.2 (regulates) ventx2.2 = 0.532670

ventx1.2 (regulates) xbp1 = 0.693715

ventx2.2 (regulates) bix1.2 = 0.066014

ventx2.2 (regulates) bmp4 = 0.349539

ventx2.2 (regulates) foxh1.2 = 0.173057

ventx2.2 (regulates) hhex = 0.083295

ventx2.2 (regulates) hnf1b = -0.083214

ventx2.2 (regulates) lhx1 = 0.012391

ventx2.2 (regulates) mix1 = -0.208376

ventx2.2 (regulates) mixer = -0.510313

ventx2.2 (regulates) msx1 = -0.156470

ventx2.2 (regulates) myc = 0.283380

ventx2.2 (regulates) sox17b.1 = 0.137830

ventx2.2 (regulates) sox21 = 0.601706

ventx2.2 (regulates) vegt = 0.000814

ventx2.2 (regulates) ventx1.2 = 0.369094

ventx2.2 (regulates) wnt11 = -0.127438

ventx2.2 (regulates) xbp1 = -0.114714

wnt11 (regulates) foxa1 = 0.070564

wnt11 (regulates) foxa2 = 0.227701

wnt11 (regulates) frzb = 0.043029

wnt11 (regulates) gata4 = -0.061933

wnt11 (regulates) gata6 = -0.005463

wnt11 (regulates) gsc = -0.051928

wnt11 (regulates) hhex = 0.214325

wnt11 (regulates) mespb = -0.024636

wnt11 (regulates) mix1 = 0.062818

wnt11 (regulates) mixer = -0.124659

wnt11 (regulates) msx1 = -0.176672

wnt11 (regulates) myc = 0.134562

wnt11 (regulates) myod1 = 0.235178

wnt11 (regulates) nodal3 = -0.035750

wnt11 (regulates) nodal6 = 0.031443

wnt11 (regulates) otx2 = -0.228568

wnt11 (regulates) sox17a = 0.030259

wnt11 (regulates) sox7 = 0.037713

wnt11 (regulates) t = 0.045364

wnt11 (regulates) vegt = 0.006791

wnt11 (regulates) ventx2.2 = -0.051491

wnt11 (regulates) xbp1 = -0.013919

xbp1 (regulates) foxa2 = -0.011271

xbp1 (regulates) foxa4a = 0.081335

xbp1 (regulates) foxh1.2 = 0.075491

xbp1 (regulates) frzb = 0.018514

xbp1 (regulates) gata4 = -0.030576

xbp1 (regulates) gata5 = 0.031104

xbp1 (regulates) gata6 = -0.091098

xbp1 (regulates) gsc = -0.153308

xbp1 (regulates) hhex = -0.065829

xbp1 (regulates) hnf1b = 0.186260

xbp1 (regulates) lhx1 = 0.172142

xbp1 (regulates) mix1 = -0.056545

xbp1 (regulates) mixer = 0.100238

xbp1 (regulates) msx1 = 0.118585

xbp1 (regulates) myc = 0.119483

xbp1 (regulates) nodal6 = 0.039143

xbp1 (regulates) otx2 = 0.541917

xbp1 (regulates) sox17a = 0.025242

xbp1 (regulates) sox21 = 0.178657

xbp1 (regulates) sox7 = -0.123654

xbp1 (regulates) vegt = 0.315482

xbp1 (regulates) ventx1.2 = 0.094168

xbp1 (regulates) wnt11 = 0.099247

**Table S2.** A complete list of the inferred (410) connections from the linear Markov model. The numbers are the inferred interaction strength.

bix1.2 (regulates) foxa1 = -0.044576

bix1.2 (regulates) foxa2 = -0.323226

bix1.2 (regulates) foxa4a = 0.117063

bix1.2 (regulates) foxh1.2 = -0.525170

bix1.2 (regulates) hhex = 0.134912

bix1.2 (regulates) msx1 = -0.072696

bix1.2 (regulates) myc = 0.015472

bix1.2 (regulates) myf5 = -0.112917

bix1.2 (regulates) sox17a = 0.064248

bix1.2 (regulates) sox21 = -0.234402

bix1.2 (regulates) vegt = 0.106287

bmp4 (regulates) ctnnb1 = 0.285919

bmp4 (regulates) foxa1 = 0.019940

bmp4 (regulates) foxa2 = 0.229213

bmp4 (regulates) foxh1.2 = 0.000189

bmp4 (regulates) frzb = -0.167995

bmp4 (regulates) gata6 = 0.099574

bmp4 (regulates) mespb = 0.395632

bmp4 (regulates) msx1 = 0.496385

bmp4 (regulates) myc = 0.694095

bmp4 (regulates) nodal3 = 0.033438

bmp4 (regulates) sox17b.1 = 0.154709

bmp4 (regulates) sox21 = 0.099468

bmp4 (regulates) sox7 = 0.165604

bmp4 (regulates) t = 0.588490

bmp4 (regulates) ventx1.2 = 0.419705

bmp4 (regulates) ventx2.2 = 0.404314

bmp4 (regulates) wnt11 = 0.043046

ctnnb1 (regulates) bix1.2 = 0.092425

ctnnb1 (regulates) bmp4 = -0.024291

ctnnb1 (regulates) foxa2 = 0.213505

ctnnb1 (regulates) foxa4a = 0.249433

ctnnb1 (regulates) foxh1.2 = -0.120747

ctnnb1 (regulates) frzb = 0.146713

ctnnb1 (regulates) gata4 = 0.134628

ctnnb1 (regulates) gata6 = 0.004946

ctnnb1 (regulates) gsc = 0.585063

ctnnb1 (regulates) hhex = 0.053003

ctnnb1 (regulates) msx1 = -0.052097

ctnnb1 (regulates) myc = -0.040433

ctnnb1 (regulates) nodal6 = 0.065953

ctnnb1 (regulates) otx2 = 0.481611

ctnnb1 (regulates) sox17a = 0.087934

ctnnb1 (regulates) sox17b.1 = 0.216540

ctnnb1 (regulates) sox7 = -0.006454

ctnnb1 (regulates) ventx1.2 = -0.122556

ctnnb1 (regulates) wnt11 = 0.061555

foxa1 (regulates) foxh1.2 = -0.532483

foxa1 (regulates) msx1 = 1.070813

foxa2 (regulates) foxh1.2 = 1.026959

foxa2 (regulates) gata6 = -0.182147

foxa2 (regulates) msx1 = -1.131715

foxa2 (regulates) myc = -0.059069

foxa2 (regulates) sox17a = -0.067878

foxa2 (regulates) sox17b.1 = -0.078455

foxa2 (regulates) sox21 = -0.136038

foxa2 (regulates) vegt = -0.044874

foxa2 (regulates) ventx1.2 = -0.105443

foxa2 (regulates) ventx2.2 = -0.153632

foxa2 (regulates) xbp1 = -0.319371

foxa4a (regulates) bmp4 = 0.067275

foxa4a (regulates) gata4 = 0.248652

foxa4a (regulates) msx1 = 0.327016

foxa4a (regulates) myc = 0.332642

foxa4a (regulates) myf5 = 0.095579

foxa4a (regulates) sox17a = -0.173603

foxa4a (regulates) sox17b.1 = -0.039825

foxa4a (regulates) t = 0.042016

foxa4a (regulates) vegt = 0.047829

foxa4a (regulates) ventx1.2 = 0.174056

foxa4a (regulates) ventx2.2 = 0.166670

foxa4a (regulates) xbp1 = 0.094179

foxh1 (regulates) bix1.2 = 0.724669

foxh1 (regulates) ctnnb1 = 0.250369

foxh1 (regulates) foxa2 = 0.020248

foxh1 (regulates) foxa4a = 0.045911

foxh1 (regulates) foxh1.2 = 0.293631

foxh1 (regulates) frzb = -0.028044

foxh1 (regulates) gsc = 0.108395

foxh1 (regulates) hhex = 0.131138

foxh1 (regulates) lhx1 = 0.052962

foxh1 (regulates) mespb = 0.004391

foxh1 (regulates) mix1 = 0.011303

foxh1 (regulates) mixer = 0.006342

foxh1 (regulates) msx1 = 0.121690

foxh1 (regulates) nodal6 = 0.133994

foxh1 (regulates) otx2 = 0.088401

foxh1 (regulates) sox17a = -0.037191

foxh1 (regulates) sox7 = 0.170320

foxh1 (regulates) vegt = 0.359971

foxh1 (regulates) ventx2.2 = 0.325555

foxh1 (regulates) xbp1 = 0.036351

foxh1.2 (regulates) bix1.2 = -0.135780

foxh1.2 (regulates) bmp4 = 0.036534

foxh1.2 (regulates) foxa4a = 0.108877

foxh1.2 (regulates) gata4 = 0.123296

foxh1.2 (regulates) gata5 = 0.557452

foxh1.2 (regulates) lhx1 = 0.035997

foxh1.2 (regulates) mixer = 0.353382

foxh1.2 (regulates) msx1 = -0.335427

foxh1.2 (regulates) myc = -0.534894

foxh1.2 (regulates) sox17a = 0.016243

foxh1.2 (regulates) sox21 = -0.008893

foxh1.2 (regulates) sox7 = -0.078955

foxh1.2 (regulates) t = 0.157641

foxh1.2 (regulates) xbp1 = 0.141030

frzb (regulates) bix1.2 = -0.033374

frzb (regulates) bmp4 = -0.208954

frzb (regulates) foxa1 = 0.082940

frzb (regulates) foxa2 = 0.098096

frzb (regulates) foxh1 = -0.057951

frzb (regulates) foxh1.2 = -0.081574

frzb (regulates) gata6 = -0.272593

frzb (regulates) gsc = -0.152737

frzb (regulates) lhx1 = -0.058594

frzb (regulates) mixer = -0.189795

frzb (regulates) msx1 = -0.458170

frzb (regulates) myc = -0.336607

frzb (regulates) myod1 = 0.142441

frzb (regulates) nodal3 = -0.045490

frzb (regulates) otx2 = -0.103431

frzb (regulates) sox17a = -0.012338

frzb (regulates) sox17b.1 = -0.395068

frzb (regulates) sox21 = 0.271575

frzb (regulates) vegt = -0.023033

frzb (regulates) ventx1.2 = -0.193896

frzb (regulates) ventx2.2 = -0.258877

gata4 (regulates) foxh1.2 = -0.835990

gata4 (regulates) msx1 = 0.328877

gata4 (regulates) sox17b.1 = -0.409227

gata4 (regulates) ventx1.2 = -0.025518

gata5 (regulates) bmp4 = 0.071118

gata5 (regulates) foxa2 = -0.262966

gata5 (regulates) foxh1.2 = 0.428034

gata5 (regulates) gata6 = 0.175470

gata5 (regulates) mespb = 0.286973

gata5 (regulates) mixer = -0.088037

gata5 (regulates) msx1 = 0.542573

gata5 (regulates) myc = 0.475550

gata5 (regulates) otx2 = -0.008671

gata5 (regulates) sox17a = 0.036921

gata5 (regulates) sox17b.1 = 0.078539

gata5 (regulates) ventx2.2 = 0.000750

gata5 (regulates) wnt11 = -0.251777

gata6 (regulates) myc = -0.679129

gsc (regulates) bix1.2 = -0.060993

gsc (regulates) ctnnb1 = 0.086753

gsc (regulates) foxh1 = -0.045400

gsc (regulates) foxh1.2 = 0.252378

gsc (regulates) frzb = 0.150460

gsc (regulates) gata4 = 0.036522

gsc (regulates) hhex = 0.177586

gsc (regulates) lhx1 = 0.631025

gsc (regulates) mespb = 0.130953

gsc (regulates) mixer = 0.096326

gsc (regulates) msx1 = -0.047023

gsc (regulates) myc = -0.215196

gsc (regulates) myf5 = 0.408610

gsc (regulates) nodal6 = -0.131143

gsc (regulates) otx2 = 0.046704

gsc (regulates) sox17a = 0.040142

gsc (regulates) sox17b.1 = 0.045566

gsc (regulates) sox21 = 0.210053

gsc (regulates) sox7 = -0.016726

gsc (regulates) vegt = 0.111710

gsc (regulates) ventx1.2 = -0.064494

hhex (regulates) bix1.2 = -0.015394

hhex (regulates) foxh1.2 = -0.184075

hhex (regulates) frzb = 0.372041

hhex (regulates) mespb = 0.015000

hhex (regulates) msx1 = -0.238580

hhex (regulates) myc = -0.120656

hhex (regulates) sox17a = 0.422095

hhex (regulates) sox17b.1 = 0.143958

hhex (regulates) sox21 = 0.140125

hnf1b (regulates) foxa1 = -0.007692

hnf1b (regulates) foxa2 = -0.336995

hnf1b (regulates) foxa4a = -0.275649

hnf1b (regulates) foxh1 = -0.095468

hnf1b (regulates) foxh1.2 = 0.005124

hnf1b (regulates) gata6 = -0.525972

hnf1b (regulates) mespb = -0.150520

hnf1b (regulates) mixer = -0.066566

hnf1b (regulates) msx1 = -0.699168

hnf1b (regulates) myc = -0.342692

hnf1b (regulates) myf5 = -0.012738

hnf1b (regulates) sox17a = -0.084365

hnf1b (regulates) sox17b.1 = -0.140734

hnf1b (regulates) sox21 = -0.468596

hnf1b (regulates) vegt = -0.043847

hnf1b (regulates) ventx1.2 = -0.166813

hnf1b (regulates) ventx2.2 = -0.204109

hnf1b (regulates) wnt11 = 0.344283

hnf1b (regulates) xbp1 = -0.108182

lhx1 (regulates) foxa4a = 0.098591

lhx1 (regulates) foxh1.2 = 0.495772

lhx1 (regulates) hnf1b = 0.033552

lhx1 (regulates) mixer = -0.061763

lhx1 (regulates) msx1 = 0.217567

lhx1 (regulates) myc = 0.221885

lhx1 (regulates) myod1 = 0.102506

lhx1 (regulates) ventx2.2 = 0.173052

lhx1 (regulates) wnt11 = -0.220216

mespb (regulates) bix1.2 = -0.002752

mespb (regulates) ctnnb1 = 0.047766

mespb (regulates) foxh1.2 = -0.136153

mespb (regulates) frzb = -0.076883

mespb (regulates) gata6 = 0.517426

mespb (regulates) lhx1 = 0.004601

mespb (regulates) msx1 = -0.207625

mespb (regulates) myc = 0.061271

mespb (regulates) sox17a = 0.310869

mespb (regulates) sox17b.1 = 0.130037

mespb (regulates) sox21 = -0.390077

mespb (regulates) wnt11 = -0.153198

mespb (regulates) xbp1 = -0.111538

mix1 (regulates) foxh1 = -0.324066

mix1 (regulates) frzb = 0.394391

mix1 (regulates) gata5 = 0.000430

mix1 (regulates) gata6 = -0.087778

mix1 (regulates) otx2 = -0.011421

mix1 (regulates) sox17b.1 = -0.022839

mix1 (regulates) sox7 = -0.222384

mix1 (regulates) t = 0.206716

mix1 (regulates) ventx2.2 = -0.096614

mixer (regulates) foxa2 = 0.546033

mixer (regulates) foxh1.2 = -0.075626

mixer (regulates) gata6 = 0.503636

mixer (regulates) hhex = 0.070097

mixer (regulates) lhx1 = 0.173321

mixer (regulates) msx1 = 0.077389

mixer (regulates) myc = 0.386304

mixer (regulates) sox17a = 0.201717

mixer (regulates) sox17b.1 = 0.545373

mixer (regulates) sox21 = -0.171510

mixer (regulates) ventx1.2 = 0.035697

msx1 (regulates) foxh1.2 = -0.107881

msx1 (regulates) myc = -0.483537

myc (regulates) foxh1 = 0.345474

myc (regulates) foxh1.2 = -0.615653

myc (regulates) gata6 = 0.138864

myc (regulates) msx1 = -0.018212

myc (regulates) sox21 = -0.021153

myc (regulates) ventx1.2 = -0.028523

myf5 (regulates) foxa2 = 0.304508

myf5 (regulates) foxh1 = -0.056543

myf5 (regulates) foxh1.2 = -0.263012

myf5 (regulates) msx1 = 0.104929

myf5 (regulates) myc = 0.181497

myf5 (regulates) myod1 = 0.991590

myf5 (regulates) sox17a = 0.010577

myf5 (regulates) sox21 = 0.524475

myf5 (regulates) sox7 = -0.019023

myf5 (regulates) vegt = -0.168075

myf5 (regulates) wnt11 = 0.883309

myod1 (regulates) foxh1.2 = 0.017237

nodal3 (regulates) foxa2 = -0.118983

nodal3 (regulates) foxh1 = 0.028257

nodal3 (regulates) foxh1.2 = -0.679732

nodal3 (regulates) frzb = -0.002621

nodal3 (regulates) gsc = 0.370499

nodal3 (regulates) mespb = 0.132388

nodal3 (regulates) msx1 = -0.038218

nodal3 (regulates) myc = -0.329000

nodal3 (regulates) nodal6 = 0.081785

nodal3 (regulates) sox17a = -0.055722

nodal3 (regulates) sox17b.1 = -0.122920

nodal3 (regulates) sox21 = -0.441361

nodal3 (regulates) sox7 = 0.022713

nodal3 (regulates) vegt = -0.179997

nodal3 (regulates) ventx1.2 = -0.159587

nodal3 (regulates) ventx2.2 = -0.263743

nodal3 (regulates) wnt11 = 0.054096

nodal3 (regulates) xbp1 = -0.275547

nodal6 (regulates) foxh1 = 0.134246

nodal6 (regulates) foxh1.2 = 0.672409

nodal6 (regulates) gsc = 0.086047

nodal6 (regulates) hhex = 0.293708

nodal6 (regulates) msx1 = 0.069804

nodal6 (regulates) myc = 0.326908

nodal6 (regulates) myf5 = -0.131807

nodal6 (regulates) nodal3 = 0.195698

nodal6 (regulates) sox21 = 0.317127

nodal6 (regulates) wnt11 = 0.045995

otx2 (regulates) bix1.2 = -0.003512

otx2 (regulates) foxa2 = 0.015982

otx2 (regulates) foxh1.2 = 0.004632

otx2 (regulates) gsc = -0.045942

otx2 (regulates) hnf1b = 0.034938

otx2 (regulates) msx1 = -0.147299

otx2 (regulates) myc = -0.010605

otx2 (regulates) nodal3 = -0.045956

otx2 (regulates) sox17a = -0.063866

otx2 (regulates) sox17b.1 = -0.000626

otx2 (regulates) sox21 = 0.088166

otx2 (regulates) ventx1.2 = -0.023579

otx2 (regulates) xbp1 = 0.094587

sox17a (regulates) foxa1 = 2.560698

sox17a (regulates) foxa2 = 2.045780

sox17a (regulates) foxa4a = 1.865924

sox17a (regulates) gata6 = 2.515061

sox17a (regulates) hnf1b = 2.464328

sox17a (regulates) myc = 0.573663

sox17a (regulates) sox21 = 2.542904

sox17b.1 (regulates) bix1.2 = -0.145416

sox17b.1 (regulates) foxa1 = 0.141401

sox17b.1 (regulates) foxa2 = -0.060927

sox17b.1 (regulates) foxa4a = -0.047083

sox17b.1 (regulates) foxh1 = -0.068453

sox17b.1 (regulates) foxh1.2 = -0.380635

sox17b.1 (regulates) gata5 = -0.077959

sox17b.1 (regulates) gata6 = -0.475155

sox17b.1 (regulates) hnf1b = 0.165959

sox17b.1 (regulates) msx1 = -0.041109

sox17b.1 (regulates) myc = -0.264275

sox17b.1 (regulates) sox17a = -0.423395

sox17b.1 (regulates) sox7 = -0.055492

sox17b.1 (regulates) wnt11 = 0.014276

sox21 (regulates) ctnnb1 = 0.190118

sox21 (regulates) foxa4a = 0.202033

sox21 (regulates) foxh1 = 0.042816

sox21 (regulates) foxh1.2 = 0.638849

sox21 (regulates) frzb = -0.049322

sox21 (regulates) gata4 = 0.082954

sox21 (regulates) lhx1 = 0.217216

sox21 (regulates) mixer = 0.003801

sox21 (regulates) msx1 = 0.035576

sox21 (regulates) myc = 0.242950

sox21 (regulates) myod1 = 0.013632

sox21 (regulates) otx2 = 0.147601

sox21 (regulates) sox17b.1 = 0.330886

sox21 (regulates) sox7 = 0.005063

sox21 (regulates) ventx1.2 = -0.169137

sox21 (regulates) xbp1 = 0.148941

sox7 (regulates) bix1.2 = 0.002166

sox7 (regulates) ctnnb1 = 0.351394

sox7 (regulates) foxa4a = 0.300925

sox7 (regulates) foxh1 = 0.104697

sox7 (regulates) foxh1.2 = 0.583340

sox7 (regulates) mespb = 0.011362

sox7 (regulates) mix1 = 0.747520

sox7 (regulates) mixer = 0.429693

sox7 (regulates) msx1 = -0.256977

sox7 (regulates) myc = -0.082741

sox7 (regulates) sox21 = 0.312502

sox7 (regulates) t = 0.088689

sox7 (regulates) vegt = 0.241422

sox7 (regulates) ventx1.2 = 0.114917

t (regulates) bix1.2 = -0.050953

t (regulates) foxa1 = 0.104938

t (regulates) foxh1.2 = -0.377586

t (regulates) msx1 = 0.278640

t (regulates) myc = -0.255247

t (regulates) myf5 = 0.328563

t (regulates) otx2 = -0.036040

t (regulates) sox7 = 0.019086

vegt (regulates) bix1.2 = 0.178821

vegt (regulates) foxh1 = 0.173523

vegt (regulates) foxh1.2 = 0.318302

vegt (regulates) frzb = 0.285305

vegt (regulates) gata4 = 0.168761

vegt (regulates) gata6 = 0.014444

vegt (regulates) gsc = -0.028859

vegt (regulates) hnf1b = 0.163790

vegt (regulates) msx1 = -0.097103

vegt (regulates) myf5 = 0.146809

vegt (regulates) otx2 = -0.399588

vegt (regulates) sox17a = 0.115810

vegt (regulates) sox17b.1 = 0.341390

vegt (regulates) sox7 = 0.080193

vegt (regulates) t = 0.136896

vegt (regulates) ventx1.2 = 0.163433

vegt (regulates) ventx2.2 = 0.160500

vegt (regulates) wnt11 = 0.214596

ventx1.2 (regulates) foxh1.2 = 0.516903

ventx1.2 (regulates) msx1 = -0.094678

ventx1.2 (regulates) myf5 = -0.316950

ventx1.2 (regulates) sox7 = 0.075298

ventx2.2 (regulates) bmp4 = 0.486204

ventx2.2 (regulates) ctnnb1 = 0.001929

ventx2.2 (regulates) foxa4a = 0.042954

ventx2.2 (regulates) foxh1 = 0.055365

ventx2.2 (regulates) foxh1.2 = 0.125765

ventx2.2 (regulates) gata4 = 0.006768

ventx2.2 (regulates) gata6 = 0.085540

ventx2.2 (regulates) mespb = 0.173438

ventx2.2 (regulates) msx1 = 0.222637

ventx2.2 (regulates) myc = 0.105983

ventx2.2 (regulates) sox17a = 0.080519

ventx2.2 (regulates) sox21 = 0.368106

ventx2.2 (regulates) vegt = 0.432251

ventx2.2 (regulates) ventx1.2 = 0.546036

ventx2.2 (regulates) xbp1 = 0.436211

wnt11 (regulates) foxh1.2 = -0.032188

wnt11 (regulates) msx1 = 0.381886

wnt11 (regulates) myc = 0.113312

wnt11 (regulates) sox17a = 0.165452

wnt11 (regulates) sox17b.1 = 0.394237

wnt11 (regulates) ventx1.2 = 0.314166

wnt11 (regulates) ventx2.2 = 0.297472

wnt11 (regulates) xbp1 = 0.213346

xbp1 (regulates) bmp4 = 0.048274

xbp1 (regulates) foxh1 = -0.002486

xbp1 (regulates) foxh1.2 = -0.352134

xbp1 (regulates) msx1 = 0.080275

xbp1 (regulates) otx2 = 0.358030

xbp1 (regulates) sox17a = 0.070736

xbp1 (regulates) sox21 = -0.039615

xbp1 (regulates) sox7 = 0.006478

xbp1 (regulates) wnt11 = 0.291074

**Table S3:** Five random networks used in the simulation of linear Markov model.

(Network I)

0 0 -2.3650 0 0 0 0 0 0 0.1086

0 -1.2492 0 0 0 -1.1464 0 0 0 0

0 0 0 0 -1.4585 0 -0.9490 0 0 0

-0.6367 0 0 0 0 0 0 0 -0.2815 0

0 0 0 0 0 -0.5466 0.4399 -0.5442 0 0

0 0 0 -0.3680 0.5375 0 0 -0.6085 0 0

0 0 0 0.6762 0 0 0 0 -0.4421 0.2888

0 -0.8776 -0.1928 0 0.0661 0 0 0 0 0

0 0 -0.3410 0 0 0 0 0 0.4510 0

0 0 0 0 1.3252 0.3873 0 0 0.8927 0

(Network II)

-0.5186 0 -0.0701 0 -1.3966 0 0 0 0 0

0 0 2.5629 0 -0.6018 0 0.0685 0 0 0

0 0 0 0 0 0.5772 0 0.8806 0 0.1486

0 0 0 0 0 0 0 -0.0777 -0.0935 -0.7084

0 -0.1015 0 0 0 0 0 0 0 1.6482

0.8661 0 0 0 0 0 0 1.1709 0 0

0 0.3824 -1.0940 0 0 0 0 0 0 -0.4745

0 0 -0.0686 0 0 0 0 -0.8482 0 0

0 0 0 1.1111 0 0 -0.0738 0 0 0

0.3020 0 0 -2.0812 0 0 0 0 0 0

(Network III)

0 0.4304 0.1502 0 0 0 0 -0.5548 0 0

0 0 0 0 0.9625 0 0 0 0 0.2469

0.4516 0 0 0 0 0 -0.5598 0.8919 0 0

0 0 0 0.2924 0 0 0.0180 0.0824 0 0

-0.5973 1.0560 0 0 0 0 0 0 0 0.4317

0.5658 0 0 0 0 -2.1888 0 0 0 0

0 0 0 0 0 0 -0.7716 0 -1.2090 0

0 0 0 0 0.0897 0.2254 1.9551 0 0 0

0 0 0 0 -0.8618 0 0 -0.8615 0 0

0 0 0.5302 0 0 0 0 0 -0.0983 0

(Network IV)

0 0 0.8639 -0.3008 0 0 0 0 0 0

0.6687 0 0 0 0 0 0 0 -0.5953 0.5916

0 -0.0306 0 0 0 0 0 -1.8984 -1.4023 0

0.2475 0 0 0 0 0 1.9569 0 0 -0.7608

1.2354 0 0 0 0 0 0 0 0 0.3369

0 0 0 0 1.0170 0 -0.0156 0 0 0

0 -0.5574 0 0 -0.1305 0 0 0 0 0

0 0 0.7276 0 0 -1.1767 0 0 0 -0.5987

0 0 0 0 0 -0.5337 0 0 0.2103 0.2294

0 0 0 0 0 0 0.7290 -0.5881 0 0

(Network V)

0 0 0.1804 0 0 0.5892 0 0 0 1.2935

0.4823 1.2670 0 0 2.0438 0 0 0 0 0

0 1.9685 0 0 0 0 0.3344 0 0 0

0 0 -0.3287 0 0 0 0 0 1.4147 0

0 0 0.0011 0 0 0 0 0.3247 0 0

0 0.6012 -0.5277 0 0 0 0 0 0.2394 0

0 0 0 -0.5211 0 0 0 0 0.1884 0

0 0 -0.7723 0 0 0 -3.0182 1.4126 0 0

-0.2454 0 0 0 0 -0.4145 0 0 0 0

0 1.1345 0 0.3538 0 -0.4252 0 0 0 0

**Table S4.** Time series data used in the simulation of linear Markov model. 15 observations are generated for each random network (Table S3) and each observation contains 4 consecutive time points. The column arrangement is in the order of time *t1* to time *t4*, observation 1 to observation 15 (i.e., totally 60 columns). Each row represents one of the 10 genes.

Time series data for network I:

Columns 1 through 10

0.7461 -0.4777 -1.0605 0.7776 -0.3130 -0.2665 0.6185 -0.8652 -0.1552 -0.2137

-0.8940 -1.0062 0.1823 -0.0165 0.3261 -0.1053 -0.2612 0.7917 -0.7772 -0.8486

0.3032 0.6918 0.1943 0.2039 0.8919 0.1008 0.4960 1.2672 0.0262 0.8014

-0.6516 -0.4797 -0.4413 -0.9127 -0.5417 -0.4104 0.7576 0.1906 -0.9833 0.6166

0.2673 -0.1418 0.1080 -0.7051 1.1115 -0.5235 0.3800 -1.0627 -0.0929 -0.1461

0.2747 0.6788 -0.6861 -1.2642 -0.1633 0.6669 -0.4282 -1.0432 -0.3132 -1.2875

0.5133 -0.8378 0.1934 -0.0603 -0.2657 0.5108 0.5530 1.0749 0.8726 -1.4066

-0.4406 0.2424 1.3159 0.2569 -0.2665 0.8836 -0.0196 -0.7096 0.8196 -0.4760

-0.1712 -0.0889 0.3688 0.6135 0.3434 -0.5429 -0.4929 -0.7417 0.0028 0.1011

0.0880 -0.2207 2.7535 0.3936 0.2367 -1.6347 -0.7559 0.6206 1.3797 -2.1356

Columns 11 through 20

-0.1151 1.4050 -1.0259 1.2413 -0.3272 -0.6930 -0.4131 0.0714 0.5015 0.9633

0.9635 -0.3784 0.4431 0.4923 -0.5647 0.5575 -0.0823 -0.7575 -1.1958 -1.0505

0.3811 -1.5501 -0.2797 -0.8914 0.9040 -0.7420 0.1316 0.3635 -0.3083 -1.1135

-0.6662 0.1708 -0.9968 0.7814 -0.4802 -0.2998 0.3767 -0.5591 0.3488 0.3375

-0.3998 -1.0707 -0.3888 0.1303 -0.4053 -0.6329 -0.2132 0.1543 0.7054 0.3156

-0.7460 -1.0620 0.0413 1.3323 -0.5151 -0.5431 -1.1959 -0.7424 -1.3652 -0.6134

0.8656 0.0597 -0.5745 -0.7699 0.1961 -1.8369 -0.8817 0.4420 -0.8332 3.2534

1.6514 -2.0497 0.2391 -0.6588 1.2226 -2.3612 -0.9797 -0.4596 0.2157 -1.0412

0.0335 2.0322 0.9278 0.2416 1.4047 1.8706 -1.6574 0.3958 -0.0870 -1.7824

3.8141 -0.9199 0.6955 -1.0206 3.6655 0.6030 -1.4108 0.2359 -1.4709 0.5102

Columns 21 through 30

0.4138 0.3447 0.4086 0.1133 0.7804 0.4085 0.7223 -0.6805 0.1284 -0.0178

-0.7115 0.0544 -0.2360 -0.1023 -0.7473 -0.2851 0.1821 0.0225 0.2410 -0.0451

-0.2770 -0.4636 0.7097 -0.9194 -0.3695 -1.0148 0.7900 -0.2098 0.4634 0.3881

-0.4272 0.1952 0.2662 -0.6094 -0.2715 -0.0622 0.2977 0.2548 0.0388 -0.7363

0.9472 0.2582 -0.5143 0.1603 0.7462 0.4044 0.0580 2.3111 0.5915 1.0223

-0.0355 -0.5140 0.3862 -0.1486 -0.0720 0.6772 0.2066 0.7272 -0.1213 -0.5330

0.3086 0.2264 -1.2995 1.1115 0.1662 -0.2140 -0.4217 -1.8952 0.3583 0.4660

-0.0153 0.0272 -0.1650 0.2482 -0.4775 -0.2585 0.9067 -1.2352 -0.3992 1.3364

-0.0245 -0.5848 0.2606 -1.4652 -1.0458 -0.5375 0.1170 -1.0418 0.1508 -0.5038

1.6416 -1.3190 -0.3983 -1.6599 -2.2988 1.1816 -1.3042 -1.0833 -1.2053 1.5462

Columns 31 through 40

0.6369 -1.3369 0.1650 1.0026 -0.7127 -0.6175 0.8226 0.6162 -0.2431 -1.5224

0.2329 0.4820 0.0868 0.3913 -0.3187 0.0330 0.0391 0.5285 -1.0421 0.2468

-0.7781 0.5758 -0.5464 -0.5867 0.0905 1.1904 -0.3244 0.9247 0.0440 1.3127

0.8539 1.2351 -0.6162 0.3282 0.2865 0.0554 -0.1089 -0.0572 0.4179 0.0975

-0.6679 0.2280 -0.4505 -0.2214 0.3792 1.0725 -0.5553 -0.6657 -0.1064 -0.6020

-0.2348 0.0252 -0.7958 -0.6191 -0.9534 2.0694 -0.2623 0.8779 -1.0610 -1.2188

0.5545 0.7733 0.4178 -0.3881 -0.7249 1.9876 0.3773 0.0269 -0.5644 -0.8145

-0.2885 -0.9610 0.0747 0.0906 -0.5318 -1.2599 0.6066 0.0872 1.0416 -0.4582

-0.6612 -0.7515 -0.0666 -0.3374 -0.0187 -0.4386 1.0589 0.8824 -1.2419 0.2202

-2.0819 -0.7269 0.8659 -2.2107 1.6364 -0.8596 1.5770 2.0501 -1.4999 0.1761

Columns 41 through 50

-0.5603 -0.7463 0.3756 -2.4480 -0.5113 0.1541 0.0548 -1.6377 -0.7416 -0.1507

-1.0422 -0.3721 -0.5605 0.4487 1.2060 0.5856 -0.8400 -1.4743 0.0454 0.1350

1.1694 -0.1111 0.2959 -0.2306 0.0720 -0.3024 -0.4371 -0.1397 -0.2002 0.7332

-1.0790 -1.0576 1.9849 1.9567 -1.0581 -0.7955 -0.7222 -1.3389 0.3920 0.2064

-0.0568 0.3964 0.3322 0.6727 0.2306 -0.9316 -0.3370 0.3981 0.4794 -0.5323

0.1746 0.4261 -0.5002 -2.0000 0.1024 0.4531 -0.5207 0.2948 -0.6444 0.6565

0.2427 1.6620 0.4639 1.4659 0.8427 -0.0979 -0.0083 0.4797 -0.5041 0.3848

0.4566 0.1324 -0.3792 -0.6296 -0.1122 -0.6757 0.1701 -0.0211 -0.0925 -0.0668

0.6260 -0.4390 -0.0838 -0.7566 -0.1778 -0.0457 -1.1253 -0.8423 0.6687 -0.4013

0.9916 0.6349 -3.5702 1.1871 -0.1063 1.3722 -1.8408 -1.4811 -2.2384 1.4405

Columns 51 through 60

-0.2730 0.3989 0.0199 -0.2904 0.5686 0.6054 -0.3391 -0.1694 -0.9646 0.2864

1.1838 1.3193 -0.6938 -0.6842 0.1052 0.6177 -1.0671 -0.1850 -0.9389 -1.4442

-0.2473 1.0357 0.2921 -0.0237 -0.2585 -0.8975 -0.5344 -0.0508 -0.8507 0.6168

2.3789 -1.4574 1.0609 0.2392 1.2050 -2.1110 0.6397 -0.0625 2.0651 -1.5202

-0.5498 -0.9762 0.1679 -0.6764 0.7643 1.1974 0.1578 -0.0191 0.4620 1.0367

-0.6206 -0.8294 0.2554 0.6683 -0.3512 0.9801 0.9563 -0.0848 -0.0577 0.5378

0.0467 -1.3515 -0.4678 -0.9868 0.1897 1.3838 -0.1975 0.5858 -0.1057 0.3665

-0.3014 -0.1412 -0.2912 0.5170 -0.6780 -0.7372 0.4050 -0.1669 -0.7779 -0.7218

0.3699 -1.1932 0.5563 0.5525 0.0173 -0.9939 0.3780 0.1740 1.4961 -0.2844

-3.6749 2.0036 -1.8488 -1.1155 -1.3195 -1.2885 -0.9847 1.8764 -2.0638 0.9387

Time series data for network II:

Columns 1 through 10

0.9449 0.6749 0.6012 0.6094 0.4457 -0.0905 0.1235 0.0120 -0.0348 -0.6319

-0.3182 0.5383 0.4749 0.5529 -1.3661 0.6296 0.5589 1.2622 -0.2578 -0.6493

0.4037 -0.2754 -0.1334 1.0076 0.0414 -0.0906 -0.6342 -1.8795 -0.5291 -0.4390

-0.4211 0.5313 0.1606 -0.3103 -0.0729 0.0431 0.1747 -0.1011 0.9307 -0.0248

0.2809 0.6223 0.1893 0.5050 0.7870 -0.6053 -0.0254 0.2059 0.1953 -0.1092

0.1601 -0.5746 0.1802 0.7503 0.3978 0.6221 -0.1141 -0.2955 0.1878 1.0706

-0.1028 -1.7778 -0.2747 -0.7504 0.4224 -0.4619 -0.0764 1.5056 0.0344 -1.5650

0.6066 0.6174 0.5818 -1.8358 -0.1797 0.2849 0.3564 1.9602 0.0458 -0.6522

0.2135 -0.3313 0.7119 -0.6414 -0.8446 -0.8018 0.2853 -0.2673 -0.5472 -1.0941

0.2036 1.3397 0.5211 0.5359 -0.1842 -0.9535 -0.2953 -0.0319 -0.3516 -0.4750

Columns 11 through 20

-0.5738 0.3313 -0.8811 -0.1514 0.2134 0.4656 -0.2127 0.0576 -0.3393 -1.4923

-0.7969 -0.9582 -0.1143 -0.2048 -1.4948 -0.2610 0.4046 0.2850 -0.1193 -0.2145

-0.3162 -0.9715 -0.1903 0.6557 0.9774 -0.2127 0.2459 0.0582 -0.2059 -0.0615

0.2048 -0.8368 -0.5571 0.4772 0.4249 -0.7957 -0.1054 -0.3511 -0.0364 -0.9732

-0.8614 -0.2782 0.0152 0.1367 -0.5413 0.1987 -0.2529 -0.0395 -0.0869 0.4986

-0.6496 1.5643 0.8814 0.1720 -0.0801 0.8566 0.4890 -0.3042 -0.3765 0.3122

-0.0053 0.2510 0.5009 -0.7168 -0.3789 0.3591 -0.6045 -0.4209 -0.9749 -0.2741

-0.3062 -0.3887 0.5509 0.7265 0.4944 0.8136 -0.0149 0.6903 -0.8410 0.0415

0.5263 0.2272 0.2319 1.2370 -0.4315 0.2051 -0.4312 0.2850 1.3865 0.1411

1.1807 -1.3267 -0.5126 -0.1125 -0.0527 0.2745 0.2681 -0.4245 0.1601 -1.3269

Columns 21 through 30

-0.3552 -0.8727 -0.1876 -0.8178 0.5910 0.3114 0.0909 -0.5312 0.0471 0.3716

-0.8821 0.1361 0.4413 0.2372 -0.5080 -0.6943 0.3735 -0.2599 0.2122 0.8772

-0.3381 -0.2752 0.0675 -1.0008 -1.3054 -0.1069 0.7030 1.6664 -0.6181 0.2371

0.4394 -0.1023 -0.1792 -3.1796 -0.1330 -0.1733 -0.5282 -1.2483 0.0538 -1.0093

0.7509 -0.0112 0.6329 2.1776 -1.0217 -0.2384 0.0072 -0.4401 0.1502 -0.4055

-0.4162 -0.1930 -0.5476 1.1815 0.7823 0.7105 -0.6642 1.7243 -0.1574 -0.5057

1.1984 -2.8666 -2.7740 -1.1917 -0.6800 -0.8614 -1.5051 1.0760 -0.0643 3.2183

-0.5358 -0.3610 0.2779 -1.4031 0.1615 0.2969 1.0055 -0.9990 -0.1990 -1.6455

1.0922 0.7148 2.3853 1.5890 0.0722 1.6618 -0.1334 0.5372 0.8545 -0.1551

-0.8976 0.1025 -0.5339 -0.7212 -0.3454 -0.5337 -0.5463 -1.7863 -0.2506 -1.3581

Columns 31 through 40

0.6169 1.9645 -0.6237 0.1430 0.0495 1.7026 1.4111 -0.5376 -0.4996 0.7552

0.3201 -0.1742 0.1143 0.6672 -0.6083 0.6329 -1.0875 0.8464 -0.3448 0.8841

0.2179 1.2246 0.3540 -0.4465 0.1859 0.2404 0.0877 -2.0010 0.8252 0.0103

-1.1192 1.2353 0.0719 -0.2744 -0.5444 -0.4411 0.2456 -0.5194 -0.6178 1.9230

-0.2865 -0.2292 0.2967 0.0949 0.6013 -0.2822 -0.4351 0.2406 0.4050 -0.9428

0.6511 0.0853 0.2748 0.6018 0.7049 -0.4635 0.1443 -0.2079 -0.2894 -0.7560

3.5794 0.5020 0.3747 0.1460 0.6561 -0.8334 0.8421 -1.8059 3.3993 -0.2931

-0.6910 0.0412 -0.6683 0.4016 0.4571 2.9775 -0.8224 -0.0343 -0.2694 -0.0682

-0.8559 -1.8998 0.3129 0.4440 -1.6087 0.0665 0.0262 -1.7204 1.2598 -2.1191

0.1911 -0.6799 0.0868 -0.1777 0.0717 0.3876 0.1316 -0.1572 0.1602 -2.8667

Columns 41 through 50

0.4131 -0.0102 -0.8151 -1.0682 -0.0708 -0.2259 1.8299 0.4524 0.6364 -0.4942

-0.3543 -1.0789 1.1648 0.9482 -0.2205 0.5126 -0.8930 0.2762 0.1926 -0.4480

-0.1206 0.0182 0.4658 0.0629 0.6710 0.0125 0.2751 -1.1113 -0.4317 -0.6995

-1.3203 -0.7328 0.2635 0.9840 0.0705 -0.1970 0.8662 0.5706 -0.4338 0.2128

0.9071 -0.1630 -0.1174 0.0881 0.6538 -0.1480 0.1006 -0.0687 0.0127 1.4788

-0.5482 -0.9115 0.2695 -0.4752 -0.0530 0.4934 0.3440 0.3858 0.8623 -0.1899

0.4412 1.5791 -4.4574 2.2977 -0.2599 -1.1369 -1.1429 -2.7555 -0.4763 1.0859

-0.3505 -0.4874 -0.3156 1.1197 -0.2824 0.0899 -1.4547 1.5002 -0.1583 -1.0354

0.3156 0.6425 -0.3206 1.7685 1.3584 -0.8193 -0.0341 2.7790 -0.2395 -0.3564

0.4822 0.3989 -0.4952 0.5565 -0.4389 0.0376 -0.0520 0.0987 0.1951 -0.0342

Columns 51 through 60

-0.3365 1.1490 0.4856 -0.4130 0.9416 0.2146 -0.1378 0.1195 0.3781 0.6574

-0.7500 0.4490 0.7367 0.6865 -0.9261 -0.5017 0.5645 -0.3233 0.0534 -2.6619

-0.1541 0.2679 -0.0049 0.9988 -0.7087 -1.7547 -0.5300 -0.5989 -0.6425 1.1784

0.7991 1.4333 -0.8613 0.5504 -0.3083 0.4207 0.2265 -0.4698 -0.7841 1.1577

-0.4794 -0.8839 -0.6951 -0.2571 -0.4396 0.0188 -0.1582 0.5899 -0.2180 0.5627

0.2101 0.2553 -0.3345 -0.3509 -0.0848 1.0199 0.2320 -0.1820 0.3359 -0.2487

0.1471 -0.0846 -0.3139 0.9374 2.8180 -0.0318 -0.4175 1.1357 1.4296 -0.0967

-0.4734 -0.4826 -0.6114 0.5239 -0.4687 -0.0364 0.3718 -0.2071 0.0196 1.0329

-1.2277 0.3050 -0.4755 -0.0363 0.3868 -1.1988 -0.1625 -1.1107 -0.2762 0.7043

0.8269 -2.4652 -0.4635 0.5136 -0.9069 0.5925 0.4307 0.0819 1.1232 -1.4305

Time series data for network III:

Columns 1 through 10

0.5611 0.8400 -0.0018 -0.5730 0.3252 -0.1767 0.2710 -2.8898 0.7702 0.0212

-0.4377 0.3244 0.9637 0.3797 0.3997 0.0535 -0.6417 -0.2058 -0.3776 -0.8235

1.2888 0.9137 -0.1461 -0.4813 0.2962 -1.1174 -0.9501 -0.1306 -0.2476 0.3526

0.5864 -0.3217 0.3097 -1.7046 0.9213 0.7588 -0.0534 0.1611 0.1231 0.2435

-0.3193 0.4480 -0.0594 0.3624 0.2125 -0.3826 -0.4903 -1.2634 -0.1050 0.0367

0.1422 0.2226 -0.4080 -1.3764 -0.8990 -0.5058 -0.4914 1.1427 -0.0957 -0.0088

-0.1853 0.6221 -0.7335 1.0481 0.3203 -0.1325 -0.3464 -1.5269 -0.1371 0.2235

0.7792 -0.8686 0.7326 1.8665 0.1885 0.3710 0.6521 1.3985 0.0885 0.5472

0.1885 -0.7086 -0.3133 -0.8933 0.7495 0.4285 0.5659 -0.3554 0.0077 0.1805

0.3902 -0.0018 0.8114 -1.6280 -0.2442 -1.1472 0.2256 -0.3716 -0.6709 0.7072

Columns 11 through 20

-0.0941 -0.8822 0.0004 -0.5547 0.2540 -0.6418 -0.6333 -0.2053 0.0247 0.5203

-0.5112 -0.1592 0.2374 -0.1686 0.7234 -1.6874 -0.4873 0.8735 0.1100 0.1701

0.2753 -0.7299 -0.9655 0.0280 0.1537 1.9660 -0.1805 0.8195 1.0250 1.5447

-0.7096 0.1444 -0.3103 -0.1286 0.9267 1.2598 -0.6327 -0.2264 -0.0788 1.9049

0.2614 -0.1391 -0.0494 -1.1743 0.1546 -1.4518 0.2393 0.3424 -0.6901 -0.7954

0.2523 1.0279 0.4909 -0.3408 0.2167 0.2051 0.7223 -0.2628 1.0229 -0.4797

-0.1099 1.4491 -0.7471 -0.0930 -0.3126 -0.1714 -1.9003 0.4077 -0.4333 0.0858

-0.3454 -0.2397 1.1285 0.0916 -0.6517 -0.4850 0.1094 -0.0575 -0.1109 1.6551

0.6045 -1.0748 -0.0009 -0.2888 0.1546 -1.7659 -0.2061 0.8002 0.2751 -1.0524

0.7072 -0.8794 -0.1135 -0.1408 0.0444 -1.3521 0.5680 0.5942 0.3963 -1.1208

Columns 21 through 30

-0.4294 -0.3688 -0.6229 -3.5640 0.5422 -0.1077 -0.0303 -0.6285 -0.4333 1.1038

-0.0158 -0.5600 -0.0363 0.0160 0.4509 -0.1992 0.0598 1.3538 0.9721 -0.1067

-0.1800 0.1522 -0.3113 -0.1994 0.1407 0.3008 -0.9755 0.0670 0.0571 -0.4670

0.0775 -0.6871 0.7403 0.6658 0.6452 0.1656 0.2516 0.0907 0.0686 -0.9572

1.1390 0.9529 0.5036 0.4890 0.4584 0.0736 0.5131 0.2106 -0.2150 -1.0532

-0.1930 0.2247 -0.9144 0.5539 -0.2963 -0.8344 -0.3403 -1.4494 -0.4279 -0.7633

-0.4546 -0.4390 0.1398 -1.3068 -0.3715 0.8222 -0.1470 0.7258 0.4923 0.9133

-0.0619 0.6390 0.0199 -1.6564 -0.3769 0.9112 0.3546 2.0963 -0.5091 0.1088

-1.5223 0.0890 0.6364 1.2826 -0.1661 -0.5124 -0.8328 0.0282 -0.6898 0.9435

0.0317 0.5478 0.7577 0.4309 0.0522 0.0928 0.4730 1.1929 0.7661 -0.1139

Columns 31 through 40

-1.2589 0.0935 0.3712 -1.2781 0.1265 -1.2680 -0.2229 -0.2201 0.2418 -0.2635

0.1082 0.4973 -0.4535 -0.2125 0.0775 0.0137 -0.4627 0.0324 -1.2455 0.0324

0.2756 -0.6902 0.0080 0.5547 0.0706 -0.8953 0.5875 -0.4430 -0.2615 -1.6713

0.0455 -0.1712 -0.0690 -0.3433 -0.1556 -0.2921 0.0093 -0.2978 -0.8419 -1.5509

0.2120 -1.4160 -0.4970 -0.1429 0.3213 -0.5182 0.6143 -0.1375 0.1354 -0.8082

0.2809 -0.5896 -0.5294 0.6297 0.1885 -1.7711 -0.0452 -0.1035 1.0571 -0.4244

-0.0788 -0.6166 0.1043 0.7102 -0.9663 -0.9970 -0.9940 -1.2019 -0.6114 0.8701

-0.3435 0.5830 -0.3824 -0.5046 -0.2995 1.2623 -0.4612 -0.5767 0.0334 0.2614

0.5924 -0.5647 -0.0651 -0.1913 -0.4665 0.7866 0.1125 0.2056 -0.2535 1.3768

-0.2415 -0.4322 0.2277 0.2525 0.0205 -1.0908 0.1673 0.0017 0.1236 0.0927

Columns 41 through 50

0.0496 0.2330 0.2496 2.3909 -0.1830 0.2433 -0.6562 0.6344 -0.0548 -1.1497

0.8863 0.5674 0.1195 1.6074 0.7936 -0.6390 -0.8790 0.5178 0.2848 -0.9498

0.2150 -0.3040 0.0211 -1.8590 -0.3780 -0.7413 -0.5840 -2.5648 -0.7533 0.2228

0.2681 -0.1895 0.5275 -0.8424 0.7607 0.0666 0.2216 -0.3198 -0.6208 -0.9876

1.1399 0.3711 0.2445 -1.3630 -0.1839 -0.2472 0.4410 0.4299 -0.7907 0.1049

-0.0189 -0.4510 0.8089 -2.2213 -0.2837 0.3227 -0.0846 0.0881 0.6439 0.7865

-0.5144 0.7525 -0.8630 0.7218 -0.3852 -0.5708 0.9624 -0.4470 0.1181 0.7359

0.6095 0.9010 0.0848 0.2169 -0.1008 0.7383 -0.9108 1.3094 -0.1346 -0.5765

-0.7582 -0.0171 -0.1219 0.9650 0.5735 -0.0485 0.2205 -0.2768 0.0946 0.1397

-0.3329 -0.8496 0.7856 -0.1353 0.1511 -1.3070 -0.5607 -0.2542 -0.8028 0.1634

Columns 51 through 60

-0.7600 -1.1046 0.2294 -0.1702 1.1202 -0.0644 0.5006 -0.3734 -0.2103 -0.2512

-0.4014 1.6057 0.4142 -0.2408 0.3921 -1.9775 -0.6847 -0.2925 -1.0255 -0.7606

0.2929 -0.0584 -0.2797 0.5696 -0.4737 -0.4328 -0.2515 -0.5265 1.5238 -0.6546

0.9663 -0.0151 -0.4935 -0.0974 0.1842 -0.3065 1.0600 0.2607 -0.5918 -0.4395

-0.3261 0.7101 0.1481 -0.2161 0.3425 -0.0360 -0.1364 0.3096 -1.2654 -1.0729

0.8458 -0.2948 0.3444 0.6047 0.2166 1.8453 0.0581 -0.3103 -0.3531 0.5039

0.4328 0.1405 0.0896 -0.4774 0.4676 -0.2161 0.8169 -0.0229 -0.6023 0.5432

-1.7440 0.6630 -0.1589 -0.1240 -0.0924 -1.2087 -0.3426 0.5616 -0.5533 -0.9319

-0.4293 0.2989 0.1667 -0.4283 -0.2254 0.6506 -0.1775 -0.3707 0.8029 -0.6689

0.1320 1.5580 -0.3923 -0.4852 1.2843 0.0514 -0.9740 -0.6425 -0.0855 0.6452

Time series data for network IV:

Columns 1 through 10

-0.9942 0.3483 -1.6778 0.1957 -0.3588 0.2307 -0.1273 -0.8018 -1.1064 -0.3877

-0.5984 -0.6926 0.2839 -1.0641 0.0774 0.3188 -0.4135 2.3511 -0.3603 -0.1335

-0.7988 0.3171 1.4959 -1.5308 -0.3658 -0.9159 0.6851 0.0136 -0.4349 -1.8517

-0.2057 -0.7214 2.4788 -0.7179 1.1026 -0.3540 0.6515 -0.6320 0.1397 -0.0949

0.8327 0.7277 0.3157 -1.2318 -0.4378 0.9162 0.4836 -0.4918 -1.2736 0.1112

0.3693 0.5647 -0.6430 1.0875 -0.4086 -1.3398 0.5027 0.2432 0.3604 -0.4006

0.0248 -0.2922 -0.1515 -1.5931 0.3365 0.1737 0.3537 1.2676 0.7529 0.1101

0.4959 0.4429 -0.7991 -0.1747 -0.0725 0.6507 0.8464 0.9775 0.3184 0.1790

0.2689 -1.6609 0.0521 0.3956 0.3756 0.1593 0.9717 -1.7672 -1.2223 -0.4748

0.2832 0.1864 0.6333 1.9141 -0.4626 -0.7410 0.1749 -1.7559 0.5005 -0.4568

Columns 11 through 20

-0.0616 -0.1591 0.3980 0.2812 0.3617 -0.2431 -0.0688 0.4827 -0.1484 0.0406

1.3477 -1.8286 0.2605 0.0256 0.3059 1.6672 -0.1468 -0.6299 0.1826 -0.1761

0.0704 -2.0527 -1.2130 0.3810 -1.0135 1.3681 0.3262 0.5886 1.8128 0.7822

-0.0093 -0.5104 -1.5860 2.3834 -3.9089 1.6461 0.6547 -1.2041 2.6900 -2.6032

0.1980 -0.0179 0.3276 0.4182 1.0137 0.9513 -0.1712 0.2656 -0.2983 0.6107

-0.1538 0.3689 -0.8499 -0.5009 0.6216 1.9554 0.2719 0.1189 1.2971 -0.9208

0.7158 -1.7459 -0.5601 -0.2398 -0.7501 0.4521 -0.9055 -0.0810 -0.3293 0.4113

-0.5745 -0.3253 -0.7349 0.5548 0.0641 -2.5779 0.7831 0.5122 -0.7113 -2.5046

0.4994 1.5834 -0.6363 -0.3927 -0.2572 1.9224 0.3674 0.3669 0.9285 -1.4268

0.1270 0.9485 -0.1172 -0.5487 0.7325 0.5141 0.1120 -1.1688 0.5241 1.9651

Columns 21 through 30

0.7504 -0.7093 0.1431 -0.0609 -1.4243 -0.0024 0.4410 -0.8675 -0.4729 0.8750

-0.6380 -0.7628 -1.0261 0.4983 0.0100 -0.1833 -0.7826 0.6204 -0.7538 -0.7074

0.3947 -0.3521 0.4498 -0.8936 -0.3321 -0.3144 0.6178 -0.6735 -1.2958 -0.0703

1.0001 0.0946 0.9371 -0.7238 -0.0186 -0.4635 1.7284 -0.7333 -0.2313 -0.5683

-1.0658 -0.1268 -1.0159 -1.1320 -0.0251 0.1667 -0.0286 0.7873 0.3551 -0.5258

0.4365 -0.3734 0.2922 -1.9274 0.0296 -0.2072 0.7292 0.0553 0.1433 0.3012

0.0280 -0.1614 0.0390 -0.2793 -0.7830 0.6464 -0.3613 1.2079 -0.4666 -0.4216

-0.3235 0.0875 0.0587 0.0314 -0.0234 -0.3687 0.3434 -0.3408 0.8796 -0.3115

0.7244 -0.0856 0.7659 -0.9348 0.1231 0.1574 -0.5810 1.0915 -0.1775 -1.1421

-0.4725 -0.2072 0.9366 1.2036 -0.3600 0.2107 0.5859 0.5436 -0.2993 0.0818

Columns 31 through 40

-0.2964 0.2591 0.2768 -0.0974 0.4484 -1.3648 -0.2422 0.1490 0.5398 -0.1345

-0.3035 -0.0434 -0.7690 -0.5272 0.5448 -0.8171 0.2686 -0.0663 0.0265 -0.9019

0.1164 -0.0121 -0.5896 -1.0294 0.7374 0.9698 1.0457 -0.8166 1.2290 -0.7613

2.4150 -0.4252 0.1144 -0.6568 2.3616 0.3637 -0.3588 -0.5424 2.0218 -1.5326

-1.2335 -0.3171 0.2210 -0.0740 0.4527 -0.7438 0.1584 -0.4251 -0.0205 -1.0145

-0.2367 -1.6876 -0.5426 -0.2087 -0.8019 0.9965 -0.1921 0.6134 0.3289 2.2539

-0.2772 -1.8172 0.1346 0.2045 -0.5503 -0.9272 0.5430 -0.6609 0.2100 0.7907

0.3806 1.4694 0.6837 -0.6004 -0.1941 0.7310 0.2852 -0.4115 -0.5016 0.7741

-0.8761 -0.4940 0.2604 0.7791 -0.6710 -0.5155 0.1350 0.4395 0.5887 -0.3778

1.3133 -1.0394 1.1268 -0.0080 0.8257 0.2002 0.1248 -0.0380 0.3024 1.1592

Columns 41 through 50

0.8635 0.3640 -0.4137 0.1687 -0.0708 0.9370 -0.0171 0.0616 -0.4860 -0.2956

-0.6235 -0.3272 -0.9209 0.6701 0.3444 -0.1405 -0.5615 1.4505 -0.6904 -0.5723

-0.6365 -0.1068 -0.1599 1.1590 0.0139 -0.8718 0.2483 1.2444 -0.6097 0.3491

-1.2456 1.1104 -1.6805 -1.3137 0.4016 0.2997 -1.3220 -0.6335 -0.2902 1.3073

0.0625 -0.2198 0.3089 1.0686 -0.2837 -0.1012 0.2233 0.0662 0.3167 0.7425

-0.2057 -0.2816 -1.1143 0.5426 0.4282 0.1794 -0.7017 0.6220 0.3286 0.6097

-0.4666 -0.2522 0.1649 1.8199 -0.4031 -0.1407 0.5574 0.5699 0.3719 -0.1165

0.9069 0.3507 -0.1593 -0.9564 -0.3437 -0.0464 -0.1157 -0.1568 0.2956 -0.0539

0.9559 0.6399 -0.2350 1.9677 0.0305 0.3599 -0.1353 -0.0845 -0.0052 -0.3662

0.9065 1.1838 -0.6552 0.9058 -0.0970 0.1286 -0.7471 0.0815 0.4290 -0.8774

Columns 51 through 60

0.6487 -2.6983 -0.3570 -0.0400 0.0406 -0.9747 0.0902 -0.2968 -0.2521 0.8727

0.8901 -1.4472 0.2063 0.7302 -0.3816 0.9577 -1.6237 -0.8147 -0.1822 1.7933

-1.8316 -0.6142 0.2991 -0.0032 -1.4072 0.5783 -0.3012 1.1480 0.5603 0.3515

-1.7966 0.7337 -0.1599 0.5242 -1.1820 -0.9575 0.5753 1.3961 -0.4429 -1.3555

-0.6397 0.9387 0.6817 -0.1420 0.8599 0.9492 -0.1198 0.3868 -1.0223 -0.7566

-0.6743 -0.1081 -0.3361 0.4689 -0.1113 0.9180 0.6075 0.2518 -0.3364 -0.3073

-0.0204 -1.8661 0.3045 0.3774 -0.2481 -2.9239 -0.3587 -0.4810 0.2272 -0.0472

-0.0414 -0.0445 -0.3877 -0.5923 -0.1847 0.6142 0.2976 -0.6532 0.0443 -0.6310

-0.1414 1.1473 0.7613 0.5320 -0.3246 0.1524 0.0148 -0.2937 -0.2297 -1.0588

-0.5551 -1.4430 0.5123 -0.0719 -0.1612 -0.5648 1.3238 0.1018 0.6438 0.1485

Time series data for network V:

Columns 1 through 10

0.0470 -0.1072 -0.1324 0.5739 -0.3689 0.3529 -1.2449 -0.2834 -0.2944 -0.1221

0.0054 0.0229 0.0531 1.0509 -0.6357 -0.2256 -0.1423 0.9106 -0.2489 0.1310

0.1219 -0.1282 0.6155 1.5249 -1.0372 -0.0405 0.9494 -0.1908 -0.1040 1.1126

0.3043 -0.1355 0.7318 3.6366 -0.2137 0.2448 0.7632 1.1617 0.1256 -1.0738

-0.8714 -0.1837 -0.3571 -0.1420 -0.9411 0.9623 0.2266 -1.6297 0.0069 -1.0821

0.1591 -0.2093 0.3197 -0.3060 1.0177 -0.1439 -0.5061 0.0800 0.3931 0.1595

0.3546 0.3825 0.1631 -1.2555 0.6481 -0.1914 -0.1716 -0.2040 0.1252 0.1215

0.1118 -0.1422 0.3121 0.4666 -1.5360 0.2371 -1.2769 1.3254 -0.3832 0.9247

0.3168 0.6029 0.4782 -0.9493 -0.8620 0.2166 -0.8686 -0.2860 0.5518 -0.3415

-0.0168 -0.2964 -0.6399 2.6759 0.6473 -0.1055 -0.1941 -0.2427 -0.3131 -0.1697

Columns 11 through 20

-0.2232 -1.3706 0.4187 0.9759 -0.7146 0.1768 0.4846 -0.2772 0.8597 -1.3590

-0.4593 1.9198 0.1661 1.0928 0.1108 0.0221 -0.7930 -0.1961 0.0465 -0.0651

-0.1102 -0.2842 -0.2114 -0.1023 -1.1418 -1.7531 -0.0564 -0.2454 0.9386 0.1920

0.3329 0.6053 0.2065 -0.4622 -0.6688 -1.1595 0.2205 0.3724 -0.1042 -0.6928

0.6878 -0.7823 0.7825 -0.3156 1.6986 -0.0747 0.4569 -0.5249 -0.1521 -0.2448

0.4708 -0.4578 -0.4583 -0.6479 0.1639 -0.2467 0.1969 -0.3084 -0.2711 -0.1021

-0.7911 -0.1412 0.0044 -1.0971 -0.7651 0.3539 0.0381 -0.1662 -0.8763 0.6583

-0.2385 1.1271 -1.0132 -0.0464 -0.0913 -0.7718 -1.1153 -0.1151 -0.2804 0.3434

0.6588 -1.9476 1.3320 -0.3555 0.5911 0.1062 -0.2229 0.5228 -1.1848 0.3108

-0.2450 -2.2275 -0.0627 -0.5812 -0.4625 0.2371 -0.9342 0.9399 0.2048 -0.8410

Columns 21 through 30

0.3300 -0.4304 -0.8018 -1.3417 0.2358 0.2011 0.3988 0.6570 -0.1871 -0.3692

-0.8953 0.4912 0.1404 -0.1704 -0.1094 -0.4712 -0.2307 1.1669 -0.6131 -0.9032

-0.2077 -0.4217 0.7092 0.1485 0.5199 -0.6595 -0.5709 -0.2183 0.3915 0.0158

0.0910 0.7635 0.2002 1.2396 -0.0573 -0.3185 -0.0226 -0.5905 0.1339 0.8651

0.5179 -0.0321 0.4343 1.5457 0.0352 -0.4515 -0.0603 0.2509 0.5882 0.4363

-0.8584 0.5131 0.7585 -0.3880 -0.0191 0.7892 -0.8884 -0.3927 1.4024 -0.8579

-0.4790 -0.4135 0.5682 -2.3616 0.0295 0.2053 0.1533 0.1251 -0.6114 0.3492

0.3131 -1.6783 0.7271 0.9971 -0.9634 1.2122 0.0193 -1.7780 0.4794 -0.0501

-0.0735 -0.6712 1.0587 1.9857 -0.2981 0.3853 -1.0606 -0.8322 0.4302 -0.6653

0.6599 -0.6836 -0.6518 0.7152 -0.0452 -0.4549 -1.3714 -0.1116 0.2865 -0.6405

Columns 31 through 40

-1.5034 0.3698 0.3832 -0.0455 0.3265 -1.0167 -0.1477 0.4823 -0.8795 0.2982

0.3652 0.8298 -0.3511 -0.5835 -0.5493 -0.1875 0.0514 0.7270 0.0397 1.3617

0.8614 -1.2107 -0.1010 -0.1023 -0.1826 -0.3647 0.0082 -0.2997 -0.2295 0.4902

1.2448 1.6967 -0.2970 -0.1413 0.2520 1.0862 1.2599 -0.0691 0.4455 -1.8665

-0.3529 -1.5201 -1.6648 0.3131 -0.5649 -1.0315 -0.4911 0.4220 1.4894 -1.1324

0.1571 0.2189 0.1542 -0.6805 0.0732 0.4191 0.9649 -1.0522 0.3334 -0.9902

-0.1674 -2.1896 -0.6795 -0.8547 -1.2982 3.9489 -1.0060 1.4611 -0.9199 0.1246

-0.3659 -0.0992 -0.4835 -0.5361 -0.1699 -0.2880 0.0874 0.6685 0.3460 0.0077

-0.8441 -0.6156 0.5606 -0.3053 -0.1611 0.8598 0.1027 0.9213 -0.8738 -0.3030

0.9150 -0.3727 -0.8591 -0.0221 0.8096 -0.7415 -0.4087 0.0836 -1.0187 -2.0406

Columns 41 through 50

0.8491 0.5480 2.7031 0.7992 -0.4952 0.0200 1.0831 0.9241 0.0331 -0.2418

0.4966 1.3116 0.0140 -0.1214 -0.8274 -0.8473 0.3057 1.8995 0.2674 -0.6302

-0.4974 0.1922 -0.8051 -0.4370 0.4650 0.3753 0.7241 0.0690 -0.4475 0.3416

1.1639 0.1918 -0.4425 -2.5287 0.2732 -0.1931 0.8851 -1.8374 0.9684 -0.3045

0.4568 0.3112 0.4111 1.5101 -0.0187 1.0132 -0.1016 1.2348 0.9043 0.4810

-0.1809 0.0489 -0.5688 0.8617 0.9466 -0.2713 -1.2985 0.5251 0.3352 0.5709

0.4317 -0.8173 -0.1636 1.4396 0.2503 0.0012 0.5281 1.7638 0.1686 0.1662

-0.4375 -0.7225 0.4763 1.8319 -0.1626 0.1638 0.0625 0.6481 -0.0852 -0.0366

0.6620 -0.4899 -0.6829 -1.3205 0.0687 0.7372 0.2201 -2.0218 0.3047 0.5846

0.1038 -0.2570 1.4605 0.3060 0.5866 0.5866 0.2303 -0.9682 -0.4620 -0.2042

Columns 51 through 60

-0.3909 0.3926 -0.0350 -0.6011 -0.4276 -1.2193 0.5825 -0.9001 0.6957 1.0656

0.1224 -1.6315 -0.4568 0.3142 0.4496 1.7303 -0.1328 -0.1087 -0.2083 -0.7011

0.9457 0.3450 0.1862 0.1290 0.3067 1.2034 -0.0256 -0.0869 0.3036 2.6437

-0.8755 -1.1690 -0.8136 -0.0617 0.9525 0.6904 -0.6268 -0.2416 1.0294 1.1689

-1.5332 -1.1516 1.2711 0.1968 -1.3733 -0.5330 -0.4222 -0.6004 0.4589 -1.0112

-0.7978 -0.2588 -0.2694 -0.0549 -0.4063 1.0437 -0.4541 -0.8622 1.1968 0.9508

-0.6350 3.0357 -0.3564 -0.4863 -0.0082 0.2850 -0.3276 0.4449 0.3959 -1.8426

0.0224 0.4580 -0.0098 -0.4369 -0.1826 0.1370 0.4822 -0.2633 0.2859 0.3471

0.6910 0.6736 0.3327 0.9246 -0.2679 -0.1815 -0.1137 -0.1448 -0.1213 -1.4541

1.1052 0.6016 0.5886 -0.5754 0.2152 -0.5311 -0.3002 -1.1557 -0.8088 -0.4091
