# Supplementary material for: Inference of the Xenopus tropicalis embryonic regulatory network and spatial gene expression patterns
Source: BMC Syst Biol. 2014 Jan 8;8:3. doi: 10.1186/1752-0509-8-3 (PMC3896677; doi:10.1186/1752-0509-8-3)
Supplement: Additional file 6 — Xenbase image data. Publication source for the spatial expression patterns of the 28 genes. [file 1752-0509-8-3-S6.docx]

1. Trindade M, Messenger N, Papin C, Grimmer D, Fairclough L, Tada M, Smith JC. (2003). Regulation of apoptosis in the Xenopus embryo by Bix3. Development. 130:4611-22.

2. Knöchel S, Schuler-Metz A, Knöchel W. (2000). c-Jun (AP-1) activates BMP-4 transcription in Xenopus embryos. Mech Dev. 98:29-36.

3. Faure S, Lee MA, Keller T, ten Dijke P, Whitman M. (2000). Endogenous patterns of TGFbeta superfamily signaling during early Xenopus development. Development. 127:2917-31.

4. Schohl A, Fagotto F. (2002). Beta-catenin, MAPK and Smad signaling during early Xenopus development. Development. 129:37-52.

5. Yost C, Torres M, Miller JR, Huang E, Kimelman D, Moon RT. (1996). The axis-inducing activity, stability, and subcellular distribution of beta-catenin is regulated in Xenopus embryos by glycogen synthase kinase 3. Genes Dev. 10:1443-54.

6. Larabell CA, Torres M, Rowning BA, Yost C, Miller JR, Wu M, Kimelman D, Moon RT. (1997). Establishment of the dorso-ventral axis in Xenopus embryos is presaged by early asymmetries in beta-catenin that are modulated by the Wnt signaling pathway. J Cell Biol. 136:1123-36.

7. Sinner D, Rankin S, Lee M, Zorn AM. (2004). Sox17 and beta-catenin cooperate to regulate the transcription of endodermal genes. Development. 131: 3069-80.

8. Sinner D, Kirilenko P, Rankin S, Wei E, Howard L, Kofron M, Heasman J, Woodland HR, Zorn AM. (2006). Global analysis of the transcriptional network controlling Xenopus endoderm formation. Development. 133:1955-66.

9. Chiu et al., manuscript in preparation.

10. Leyns L, Bouwmeester T, Kim SH, Piccolo S, De Robertis EM. (1997). Frzb-1 is a secreted antagonist of Wnt signaling expressed in the Spemann organizer. Cell. 88:747-56.

11. Wang S, Krinks M, Lin K, Luyten FP, Moos M Jr. (1997). Frzb, a secreted protein expressed in the Spemann organizer, binds and inhibits Wnt-8. Cell. 88:757-66.

12. Weber H , Symes CE , Walmsley ME , Rodaway AR , Patient RK . (2000). A role for GATA5 in Xenopus endoderm specification. Development. 127:4345-60.

13. Afouda BA, Ciau-Uitz A, Patient R. (2005). GATA4, 5 and 6 mediate TGFbeta maintenance of endodermal gene expression in Xenopus embryos. Development. 132:763-74.

14. Cho KW, Blumberg B, Steinbeisser H, De Robertis EM. (1991). Molecular nature of Spemann's organizer: the role of the Xenopus homeobox gene goosecoid. Cell. 67:1111-1120.

15. Jones CM, Broadbent J, Thomas PQ, Smith JC, Beddington RS. (1999). An anterior signalling centre in Xenopus revealed by the homeobox gene XHex. Curr Biol. 9:946-54.

16. Taira M, Otani H, Saint-Jeannet JP, Dawid IB. (1994). Role of the LIM class homeodomain protein Xlim-1 in neural and muscle induction by the Spemann organizer in Xenopus. Nature. 372:677-9.

17. Sudou N, Yamamoto S, Ogino H, Taira M. (2012). Dynamic in vivo binding of transcription factors to cis-regulatory modules of cer and gsc in the stepwise formation of the Spemann-Mangold organizer. Development. 139:1651-1661.

18. Skirkanich J, Luxardi G, Yang J, Kodjabachian L, Klein PS. (2011). An essential role for transcription before the MBT in Xenopus laevis. Dev Biol. 357:478-91.

19. Suzuki A, Ueno N, Hemmati-Brivanlou A. (1997). Xenopus msx1 mediates epidermal induction and neural inhibition by BMP4. Development. 124:3037-44.

20. Smith WC, McKendry R, Ribisi S Jr, Harland RM. (1995). A nodal-related gene defines a physical and functional domain within the Spemann organizer. Cell. 82:37-46.

21. Khokha MK, Yeh J, Grammer TC, Harland RM. (2005). Depletion of three BMP antagonists from Spemann's organizer leads to a catastrophic loss of dorsal structures. Dev Cell. 8:401-11.

22. Zamparini AL, Watts T, Gardner CE, Tomlinson SR, Johnston GI, Brickman JM. (2006). Hex acts with beta-catenin to regulate anteroposterior patterning via a Groucho-related co-repressor and Nodal. Development. 133:3709-22.

23. Blitz IL, Cho KW. (1995). Anterior neurectoderm is progressively induced during gastrulation: the role of the Xenopus homeobox gene orthodenticle. Development. 121:993-1004.

24. Pannese M, Polo C, Andreazzoli M, Vignali R, Kablar B, Barsacchi G, Boncinelli E. (1995). The Xenopus homologue of Otx2 is a maternal homeobox gene that demarcates and specifies anterior body regions. Development. 121:707-20.

25. Zorn AM, Butler K, Gurdon JB. (1999). Anterior endomesoderm specification in Xenopus by Wnt/beta-catenin and TGF-beta signalling pathways. Dev Biol. 209:282-97.

26. Smith JC, Price BM, Green JB, Weigel D, Herrmann BG. (1991). Expression of a Xenopus homolog of Brachyury (T) is an immediate-early response to mesoderm induction. Cell. 67:79-87.

27. Gawantka V, Delius H, Hirschfeld K, Blumenstock C, Niehrs C. (1995). Antagonizing the Spemann organizer: role of the homeobox gene Xvent-1. EMBO J. 14:6268-79.

28. Onichtchouk D, Gawantka V, Dosch R, Delius H, Hirschfeld K, Blumenstock C, Niehrs C. (1996). The Xvent-2 homeobox gene is part of the BMP-4 signalling pathway controlling dorsoventral patterning of Xenopus mesoderm. Development. 122:3045-53.

29. Carron C, Bourdelas A, Li HY, Boucaut JC, Shi DL. (2005). Antagonistic interaction between IGF and Wnt/JNK signaling in convergent extension in Xenopus embryo. Mech Dev. 122:1234-47.

30. Cao Y, Knöchel S, Oswald F, Donow C, Zhao H, Knöchel W. (2006). XBP1 forms a regulatory loop with BMP-4 and suppresses mesodermal and neural differentiation in Xenopus embryos. Mech Dev. 123:84-96.
